# Evaluation of potential role of R-loop and G-quadruplex DNA in the fragility of c-*MYC* during chromosomal translocation associated with Burkitt’s lymphoma

**DOI:** 10.1016/j.jbc.2023.105431

**Published:** 2023-11-04

**Authors:** Nitu Kumari, Kohal Das, Shivangi Sharma, Sumedha Dahal, Sagar Sanjiv Desai, Urbi Roy, Anju Sharma, Meghana Manjunath, Vidya Gopalakrishnan, S.T. Retheesh, Saniya M. Javadekar, Bibha Choudhary, Sathees C. Raghavan

**Affiliations:** 1 Department of Biochemistry, Indian Institute of Science, Bangalore, India; 2 Institute of Bioinformatics and Applied Biotechnology, Bangalore, India; 3 Department of Zoology, St Joseph's College, Irinjalakuda, Kerala, India

**Keywords:** RNA-DNA hybrid, t(8;14) translocation, AID, cytidine deaminase, genomic instability, G-quartets, RNase H, DNA double-strand break, R-loop, chromosomal aberration

## Abstract

t(8;14) translocation is the hallmark of Burkitt’s lymphoma and results in c-*MYC* deregulation. During the translocation, *c-MYC* gene on chromosome 8 gets juxtaposed to the Ig switch regions on chromosome 14. Although the promoter of c*-MYC* has been investigated for its mechanism of fragility, little is known about other c*-MYC* breakpoint regions. We have analyzed the translocation break points at the exon 1/intron 1 of c-*MYC* locus from patients with Burkitt’s lymphoma. Results showed that the breakpoint region, when present on a plasmid, could fold into an R-loop confirmation in a transcription-dependent manner. Sodium bisulfite modification assay revealed significant single-strandedness on chromosomal DNA of Burkitt’s lymphoma cell line, Raji, and normal lymphocytes, revealing distinct R-loops covering up to 100 bp region. Besides, ChIP-DRIP analysis reveals that the R-loop antibody can bind to the breakpoint region. Further, we show the formation of stable parallel intramolecular G-quadruplex on non-template strand of the genome. Finally, incubation of purified AID *in vitro* or overexpression of AID within the cells led to enhanced mutation frequency at the c-*MYC* breakpoint region. Interestingly, anti-γH2AX can bind to DSBs generated at the c-*MYC* breakpoint region within the cells. The formation of R-loop and G-quadruplex was found to be mutually exclusive. Therefore, our results suggest that AID can bind to the single-stranded region of the R-loop and G4 DNA, leading to the deamination of cytosines to uracil and induction of DNA breaks in one of the DNA strands, leading to double-strand break, which could culminate in t(8;14) chromosomal translocation.

Chromosomal rearrangements are one of the major threats to the cell and hence to human health (1, 2, 3). Such genomic rearrangements result in structural alteration of the genome through the joining of normally distant sequences. Chromosomal translocations are one of the most common genomic rearrangements in cancers (4, 5). Among different types of cancers, recurrent translocations are frequently observed in the case of hematological malignancies, namely, lymphoma and leukemia (6, 7). These rearrangements involve the exchange of parts between nonhomologous chromosomes, which majorly leads to two outcomes contributing to oncogenesis (8, 9, 10). The first involves the fusion of two genes to produce a chimeric protein with oncogenic activity. A typical example is the Philadelphia chromosome found in a subtype of acute lymphoblastic leukemia (Ph^+^ ALL) and chronic myeloid leukemia (CML), in which the BCR-ABL fusion gene encodes a protein with deregulated kinase activity (7, 11, 12, 13). Second, when *cis*-regulatory transcriptional elements from one gene are juxtaposed to a proto-oncogene, thereby causing aberrant expression of the growth-promoting oncogene, such as in Burkitt’s lymphomas (BLs) (7, 14, 15, 16).

BL is a highly aggressive B cell neoplasia, characterized by the presence of specific reciprocal chromosomal translocations involving the c-*MYC* oncogene on chromosome 8 and the immunoglobulin gene on chromosome 14 (17, 18, 19). This translocation results in the juxtaposition of the c-*MYC* gene on chromosome 8 to the *IgH* locus on chromosome 14, bringing c-*MYC* under the regulation of the *IgH* enhancer (20), leading to deregulated expression of the *c-MYC* gene, finally contributing to cellular transformation. Although *c-MYC/IGH* rearrangements drive both sporadic BL (sBL) and endemic BL (eBL) (21), the precise location of the breakpoints differs in the two subsets (16, 22, 23, 24). In sBLs, translocations occur within the c-*MYC* exon 1 – intron 1 region, whereas eBL translocations are noncanonical in that they map hundreds of kilobases upstream from c-*MYC* basal promoters (22).

Formation of paired double-strand breaks (DSBs) on separate chromosomes, followed by the proximity of the broken ends and subsequent joining of the heterologous DNA ends are the basic requirements for the generation of chromosomal translocation (3, 6, 8, 25). Two major mediators proposed for chromosomal translocations are RAGs (recombination activating genes) owing to its sequence and structure-specific nuclease activity and AID (activation induced cytidine deaminase) which deaminates the cytosines present in single-stranded (ss) DNA regions into uracil (5, 6, 10, 26). Since the action of both of these mediators is dependent on DNA sequence, structure, or both, they play an important role in the maintenance of genomic integrity (5). Though the Watson-Crick paired B-form DNA is mostly adopted by the genome, there are several forms of non-B DNA described, including Z-DNA, H-DNA (triplex DNA), cruciform DNA, tetraplexes (G-quartets), and RNA-DNA hybrid (R-loops) (9, 27, 28, 29, 30, 31).

Previous studies have implicated H-DNA formation at *c-MYC* promoter regions (32). Studies from our lab have shown the role of G-quadruplexes in the fragility of *HOX11* and *BCL2* breakpoint regions during chromosomal translocations associated with T-cell leukemia and follicular lymphoma, respectively (4, 7, 19, 29, 33). Among other forms of alternate DNA structures, R-loops have increasingly gained attention over the last decade and have been implicated in genomic instability (34). R-loops are more susceptible to DNA damage owing to the presence of long ssDNA; thus, they are prone to transcription-associated mutagenesis (TAM), recombination (TAR), and DSBs (34, 35, 36, 37). Previous studies have also shown that G-quadruplex (G4) structures can be formed on the nontemplate strand of R-loop due to negative supercoiling and high transcription rate on the template DNA (38).

Studies have described the *in vivo* role of R-loop formation in specific physiological events. For example, R-loop formation in activated B-lymphocytes has been shown to promote deamination of cytosine during immunoglobulin class switch recombination (CSR) (34). Interestingly, R-loop structure formation also plays a role in regulating transcription. It is known to facilitate the binding of transcription factors as well as RNA Pol II pausing. R-loop mediated RNA pol II pausing at transcription terminator elements induces anti-sense transcription resulting in the formation of dsRNA, which activates RNA interference (RNAi) and histone modification machinery leading to the formation of repressive chromatin marks (39).

AID is a cytidine deaminase that was described several years back (40). The role of AID in generating the DNA breaks during CSR and somatic hypermutation (SHM) is well established (10, 26). Recent studies have also suggested the role of AID in the generation of *c-MYC/IGH* translocation (24, 41, 42). However, it was intriguing that the breakpoints of translocations in sporadic BL are widely distributed upstream and downstream of the c-*MYC* promoter.

The promoter region of the c-*MYC* gene has been investigated for its potential to form altered DNA structures; however, the basis for DNA breaks at other regions of the c-*MYC* gene was largely unknown. Here, we investigated the causes of fragility downstream of promoter P2. As AID is speculated to be a major player in c-*MYC/IGH* translocation, the single-strandedness of these breakpoint regions is a prerequisite. Thus, we wondered whether non-B DNA structures could form a basis for transcriptionally active c-*MYC* exon 1-intron 1 region. We mapped all the reported c*-MYC* breakpoints and scored for the formation of non-B DNA structures in the breakpoint clusters. Interestingly, we found that a portion of the c-*MYC* breakpoint region can form R-loops with lengths ranging from 50 to 100 nt. We demonstrated the single-strandedness of the region using multiple assays and found that an R-loop can be formed in both the template and the non-template strands. Besides, we observed the formation of two independent G-quadruplex structures in the non-template strand of the R-loop forming region in a transcription-independent manner. ChIP sequencing and other biochemical and in-cellular studies showed that AID can bind to these c-*MYC* breakpoint regions leading to the generation of DSBs at the c-*MYC* breakpoint region. Although our results show the formation of R-loop and G-quadruplexes as independent events, there is a possibility that these structures may coexist *in vivo*. Thus, our study delineates mechanisms of fragility at the *c-MYC* during t(8;14) translocation.

## Results

### Analysis of c-*MYC* translocation breakpoint region reveals two major clusters

**Figure 1:**
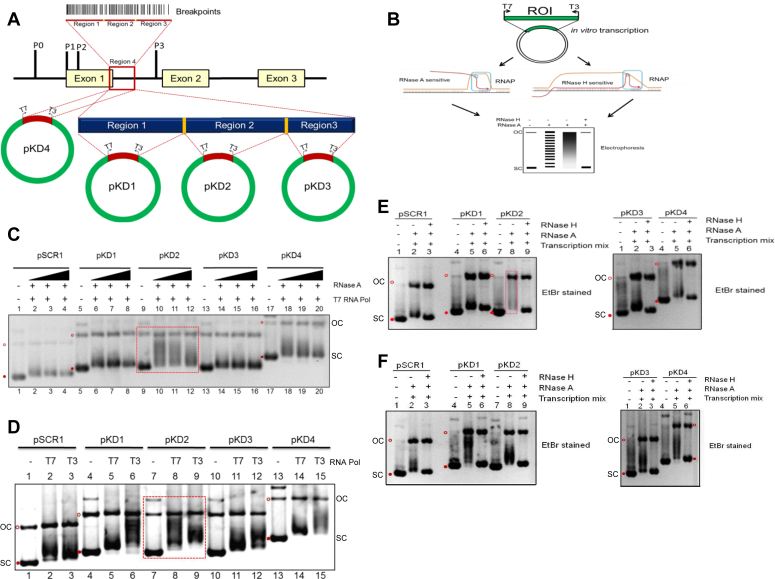
**Evaluation of formation of R-loop structures at c-*MYC* breakpoint region associated with Burkitt’s lymphoma.***A*, schematic representation showing human *c-MYC* gene, which contains four promoters (P0, P1, P2, and P3), three exons (exon 1, 2, and 3), and two introns. The *vertical lines* indicate c-*MYC* breakpoints reported during t(8;14) translocation in Burkitt’s lymphoma. The breakpoint region (934 bp) covering portions of exon 1 and intron 1 used for this study is labeled as Region 4 (indicated by the *red box*) and cloned into pBS (SK+) to generate pKD4. Region 4 is subdivided into three regions, named Region 1, Region 2, and Region 3, which were cloned into pBS (SK+), generating pKD1, pKD2, and pKD3. *B*, schematic representation of the assay used for the detection of R-loops. Plasmids containing regions of interest (ROIs) are transcribed using T7 or T3 RNA polymerase. The reaction products were resolved on an agarose gel following treatment with RNase A or RNase H. R-loops are RNase H sensitive, whereas they are resistant to RNase A, resulting in the shift indicated in the gel. *C*, pKD1, pKD2, pKD3, pKD4, and pSCR1 were transcribed *in vitro* using T7 RNA polymerase; the reaction was heat-inactivated and treated with increasing order of RNase A (100, 300, and 500 ng), resolved on an agarose gel, and stained with ethidium bromide after electrophoresis. Open *red circle* (OC) denotes *open circular*, and filled *red circle* denotes supercoiled (SC) plasmid. *D*, pKD1, pKD2, pKD3, pKD4, and pSCR1 were transcribed *in vitro* with T7 (physiological orientation) and T3 RNA polymerase (anti-physiological orientation). For other details, refer to panel *C*. *E* and *F*, R-loop formation in physiological orientation is analysed for pSCR1, pKD1, pKD2, pKD3, and pKD4 following RNase H digestion. The plasmid substrates were either mock transcribed (lanes 1, 4, and 7 for the left panel and lanes 1 and 4 for the right panel) or transcribed with T7 RNA polymerase (E) or transcribed with T3 RNA polymerase (F) and treated with RNase A (lanes 2, 5, and 8 for the left panel and lanes 2 and 5 for right panel). Lanes 3, 6, and 9 (for left panel) and lanes 3 and 6 (for right panel) represent transcribed plasmids treated with RNase A and RNase H. R-loop molecules run slower than SC and are observed as a shifted species as a smear (*boxed*).

t(8;14) translocations have been observed in almost 80% of BLs reported so far (43, 44). DNA sequencing studies revealed precise translocation breakpoint junctions in several cases of t(8;14). Since we were interested in understanding the mechanism of the fragility of the c-*MYC* region, previously reported breakpoints of t(8;14) in Burkitt’s lymphoma were mapped onto human chromosome 8 genomic contig, GRCh37.p10 Primary Assembly (NCBI Reference Sequence: NT_008046.16) (43, 44). Results showed that breakpoints from 143 patients were spread across a ∼4 Kb region, including promoters (P1 and P2), first exon, and first intron (Fig. S1*A*). Interestingly, within this region, we observed two breakpoint clusters, which were named Cluster I and II (Fig. S1*A*). The breakpoints mapped in the region around promoters were named Cluster I (Fig. S1*A*). In the present study, we investigated the mechanism of fragility at Cluster II, spanning a region of 934 bp within exon 1/intron 1 of *c-MYC*, covering 66 patient breakpoints (Fig. 1*A*).

For the convenience of analysis, we further divided Cluster II into three, namely, Region 1 (350 bp), Region 2 (331 bp), and Region 3 (293 bp) (Fig. S1*B*). DNA sequence from each region was analyzed *in silico* for various parameters such as the presence of non-B DNA motifs, AID binding WRC motifs (where W = A/T, R = purine, and C = cytosine), etc. Results showed 25, 15, and 11 WRC motifs in Region 1, Region 2, and Region 3, respectively. Further, potential non-B DNA motifs were evaluated using Non-B DB v2.0 (30). Results showed a single G-quadruplex motif in Region 1, whereas Region 3 contained very short cruciform motifs (Fig. S1*C*), however, this may not be sufficient to explain the breakpoints observed in those regions. There were no non-B DNA motifs observed in Region 2, although it encompassed a considerable amount of patient translocation breakpoints (Fig. S1*C*). While the GC content of Regions 1 and 3 was high (57%), Region 2 showed G skewness. Previously, it has been shown that guanine-rich non-template strands at the class switch regions can form R-loops during transcription (45). Therefore, we wondered whether these fragile regions could fold into an RNA-DNA hybrid in a transcription-dependent manner.

### RNA-DNA hybrid is formed at cluster II of the *c-MYC* breakpoint region in a transcription-dependent manner

In order to investigate potential RNA-DNA hybrid formation at Cluster II, we resorted to standard *in vitro* transcription assays (Fig. 1, *A* and *B*). RNA-DNA hybrid formation on plasmid results in paired two-stranded hybrid DNA structure, wherein the nascent RNA is hybridized to the template strand and the non-template DNA strand remains as a loop due to which it is also named as R-loop (35, 37) (Fig. 1*B*). RNA-DNA hybrid is sensitive to RNase H, while resistant to RNase A.

Firstly, each region (Regions 1, 2, 3, and 4) was cloned into multiple cloning sites of pBlueScript SK+, as it possesses T3 and T7 promoters (Fig. 1*A*). The identity of the resulting plasmids, pKD1, pKD2, pKD3, and pKD4, was confirmed by DNA sequencing. Plasmids were then subjected to R-loop analysis following *in vitro* transcription and treatment with various concentrations of RNase A (Fig. 1*C*). Transcription was carried out either in physiological (T7 RNA Polymerase) or anti-physiological orientation (T3 RNA Polymerase), and the products were analyzed on an agarose gel (1%) following post-staining with ethidium bromide (Fig. 1, *D*–*F*). Results showed a distinct mobility shift due to a variety of conformational isomers in the case of pKD2 when transcribed using T7 RNA Pol, while such a shift was not evident in the case of pKD1, pKD3, and pKD4, and was comparable to negative control, pSCR1, a plasmid containing *BCL2* region, unable to form RNA-DNA hybrids (Fig. 1, *C* and *D*). Based on multiple RNase A titration assays, a concentration of 90 ng/μl was used for further studies unless otherwise specified. Interestingly, we observed a pronounced mobility shift due to partially relaxed conformational isomers (as the linking number is unchanged) in the case of pKD1, pKD3, and pKD4, when transcribed using T3 RNA Pol, which was absent in the case of pSCR1 (Fig. 1*D*). Unlike in the case of pKD2, where the transcription was in physiological orientation, transcription in anti-physiological orientation resulted in partial relaxation. Among the plasmids, maximum relaxation was seen in the case of pKD1 (covering Region 1), which suggests the possibility of R-loop formation in anti-physiological orientation (Fig. 1*D*). These results reveal the potential RNA-DNA hybrid formation in pKD2 upon transcription in a physiological direction. In contrast, pKD1, pKD3, and pKD4 showed RNA-DNA hybrid formation in anti-physiological orientation.

The shift in the position of supercoiled DNA upon transcription in both orientations is due to the positive supercoiling in the direction of progression that leaves the region of negatively supercoiled DNA behind. This generates multiple conformational isomers of the plasmid, explaining the shift in the position. It is well known that rG:dC pairing has maximum thermodynamic stability and that in turn stabilizes the R-loop (46). Thus, molecules with such pairing in long stretches (R-loops) may show a complete shift on an agarose gel, in contrast to less shift displayed by other conformational isomers (46).

To test whether the gel mobility shift observed following transcription at Region 1, 2, and 4 in the above studies was indeed due to RNA-DNA hybrid formation, we employed RNase H digestion (12 U), which is known to digest RNA-DNA hybrids. Agarose gel electrophoresis showed that the specific shift observed following transcription in physiological orientation in the case of pKD2 was reverted upon RNase H digestion (Fig. 1*E*, lane 9), whereas such a complete shift was absent in the case of other plasmids. Interestingly, the shift observed in the reverse orientation in the case of Regions 1, 3, and 4 were also digested by RNase H, suggesting the presence of transcription on anti-sense or template strand in an anti-physiological direction (Fig. 1*F*).

### Bisulfite modification assay reveals short single-stranded DNA regions at a single molecule level within the genome of Raji cells

Bisulfite modification assay is a technique useful for scoring single-strandedness present on the genome due to the formation of non-B DNA structures at a single molecule level (4, 34, 47). Sodium bisulfite can react with cytosine when present on a single-stranded DNA, converting it to uracil. Since non-B DNA structures such as RNA-DNA hybrid and G-quadruplex contain ssDNA at the complementary strands (3, 7), this assay can be used as a tool to detect the presence of such structures in the genome. To check the single-strandedness at *c-MYC* region covering translocation breakpoints, genomic DNA was isolated using a nondenaturing method from Raji cells, which is derived from a Burkitt lymphoma patient and subjected to sodium bisulfite modification assay.

**Figure 2:**
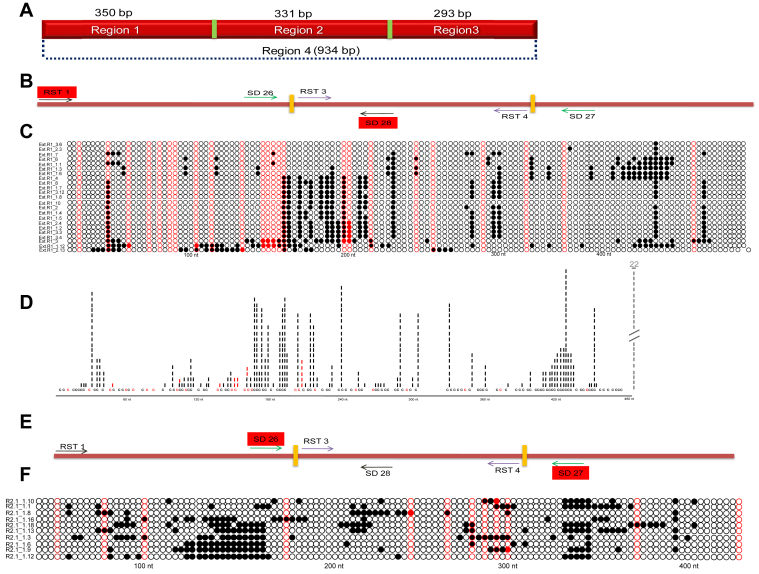
**Bisulphite modification analysis for single-stranded regions in c-*MYC* gene in the genome.***A*, schematic representation of the 934 bp breakpoint region covering portions of exon 1 and intron 1 used for the study (Region 4). Region 4 is subdivided into Region 1, Region 2, and Region 3 (See Fig. 1*A* for details). *B*, schematic representation for primer binding sites of RST1 and SD28, which amplifies Region 1 and overlapping sequences from Region 2 (amplicon size of 478 bp). *Yellow rectangles* are used to divide the regions. *C*, representative distribution of bisulfite-converted cytosines in the bottom strand of extended Region 1 (478 bp). The region of interest was PCR amplified from the bisulfite-treated genomic DNA from Raji cells, TA cloned, and sequenced. A single DNA molecule (a clone) is represented in each row. Fourty eight clones were sequenced for extended Region 1, out of which 26 were from the bottom strand and are represented. The *filled black circle* indicates cytosine conversion to uracil, while the *open circle* indicates no conversion. CpG sites are marked with a *red circle*, CpG sites present after two continuous conversions are considered as converted cytosines and marked with a *filled red circle*, while an *open red circle* indicates a CpG site without conversion. *D*, depiction of cumulative frequency of conversion of cytosine to uracil on genome due to R-loop formation at extended Region 1 (478 bp). Fourty eight clones were sequenced for extended Region 1, out of which 22 were from the top strand and are represented in the figure. *Vertical dashes* (*vertical bars*) indicate the sensitivity for the top strand, and each *vertical bar* represents a cytosine conversion on one molecule. *E*, schematic representation for primer binding sites in *c-MYC* Region 2. In this case, Region 2 is amplified using primer set, SD26 and SD27 (amplicon size of 414 bp). *F*, representative bisulfite sensitivity for the bottom strand of 414 bp region spanning Region 2. Each row of circles represents a single DNA molecule (a clone). A total of 56 clones were sequenced for Region 2 using primer sets SD26 and SD27. Ten selected clones out of 25 from the bottom strand are shown for representation. For other details, refer to panel *C* legend.

The above *in vitro* transcription studies suggest the formation of R-loop in an anti-physiological direction when *MYC* breakpoint Region 1 was studied, while the R-loop formation was in a physiological direction when Region 2 was studied. The formation of such structures within the genome will lead to single-strandedness in the complementary strands within the regions. To evaluate this, following the bisulfite modification assay, regions of interest were PCR amplified, cloned, and sequenced (Fig. 2, *A* and *B*). Analysis of 48 DNA molecules from extended Region 1 (478 bp; Fig. 2, *A* and *B*) comprising a portion of Region 2 in addition to Region 1 showed stretch conversion of C→T in the bottom strand or template strand of several clones (Fig. 2, *B* and *C*), suggesting R-loop formation in anti-physiological orientation, which was consistent with gel mobility shift assays (Fig. 1*D*). Among 22 clones from the bottom strand, 13 had a continuous conversion, which covered 50 to 65 nt at multiple regions (Fig. 2, *C* and *D*). Analysis of converted molecules in extended Region 1 revealed two different peaks of single-stranded regions. However, one of the single-stranded regions matched with the G4 motif in the complementary strand (Fig. 2, *C* and *D*; positions, 400–450 nt), while the other peak of the single-stranded region suggests the formation of R-loops in the anti-physiological direction at Region 1. Importantly, such stretches of single-strandedness were absent when the top strand or non-template strand was analyzed (Fig. S2, *B* and *C*). The only molecule that showed stretch conversion spanned to a region of only 31 nt out of 478 nt region (Fig. S2*C*). In summary, our results suggest the formation of short R-loops in anti-physiological orientation in the genome (Figs. 2, *A*–*D* and S2, *A*–*D*), which was consistent with *in vitro* transcription assays (Fig. 1, *C*–*F*). When CpG sites are present after four continuous conversions of cytosine, such ^me^CpGs are considered present in the single-stranded region of the DNA.

To investigate whether the single-strandedness seen at Region 1 of *c-MYC* Cluster II inside the genome of Raji cells was indeed transcription-dependent, Raji cells were treated with actinomycin D (0.5 μM) to block the transcription (12 h) (48, 49). Cells were harvested, chromosomal DNA was isolated using the nondenaturing method and subjected to bisulfite modification assay followed by PCR, cloning, and sequencing of extended Region 1. Although 60 clones were sequenced (35 bottom and 25 top), none of the clones showed continuous stretch conversion of >10 cytosines (Fig. S3, *A*–*E*). The observed moderate conversions were random, though the bottom strand still had an overall higher conversion frequency than the top strand (Fig. S3, *B* and *C*). These results suggest that the single-strandedness observed following bisulfite modification was indeed transcription-dependent and could be due to R-loop formation inside the Raji cells.

The gel shift assays also revealed the formation of RNA-DNA hybrids in the physiological orientation (Fig. 1, *D*–*F*). Based on this, Region 2 of the *MYC* breakpoint region was analyzed for R-loop formation in the genome. To do this, bisulfite-treated genomic DNA was analyzed using PCR primers specific to Region 2, followed by DNA sequencing. Among 110 clones sequenced, 70 were derived from the bottom strand, while 40 were from the top strand (Fig. S2, *E*–*G*). If the R-loop formation is at physiological orientation, one will anticipate C→T conversion on the top strand or non-template strand, whereas if C→T conversion is on the bottom strand, it suggests the R-loop formation in anti-physiological orientation. Analysis revealed no stretch conversion of cytosines in the top strand (Fig. S2*F*) compared to the bottom strand. In the case of the bottom strand, 11 out of 70 molecules (15.7%) showed stretch conversion of 12 cytosines when a region of 15 cytosines was considered (Fig. S2*G*). This reveals that single-strandedness is of shorter length and covers a 30 to 40 nt length region. However, we observed two stretches of single-strandedness in this region (Fig. S2*G*). Closer analysis of these stretches suggests that the complementary sequence of the single-stranded (ss) region possesses potential G-quadruplex forming motifs (around 80–100 nt and 270–300 nt), although they did not follow the pattern of conventional G4 motifs (Fig. S2*G*). Importantly, no stretch conversions were observed when top-strand DNA (40 clones) was analyzed (Fig. S2*F*). Further, to rule out the possibility of primer biases during PCR, another set of primers specific for Region 2 was used (amplicon size of 414 bp) for bisulfite sequencing (Figs. 2, *E* and *F* and S2, *H* and *I*). When 56 clones were analyzed, 31 were derived from the top strand, while 25 were from the bottom strand. Results showed only limited stretch conversion of cytosines in the top strand as compared to the bottom strand (Figs. 2*F* and S2*I*). Further, among 25 bottom strand derived molecules, 3 (clone no. R2.1_1.6, R2.1_1.9, and R2.1_1.12) of them showed stretch conversion of 13 or more cytosine (12%, only selected clones with continuous cytosine conversions are shown in the figure) (Fig. 2*F*). As seen above, the ss region was of 30 to 40 nt length complementary to unconventional G-quadruplex motifs. These results suggest that although we were able to detect a short ss region within the genome, we were unable to detect the formation of the R-loop when Region 2 was analyzed. In short, although short R-loop formation was seen in an anti-physiological direction, we could not find any evidence for the formation of R-loop in physiological orientation in the genome of Raji cells. However, bisulfite modification assay on plasmids following *in vitro* transcription in physiological orientation resulted in C→T conversion in the top strand, confirming the formation of R-loop in the sense strand or non-template, which was consistent with the results obtained during gel shift assay (Fig. S2*J*).

These results are in line with our hypothesis that transcription at the *MYC* breakpoint region occurs in the anti-physiological direction, and therefore, the single-strandedness is observed in the bottom strand of genomic DNA at extended Region 1. This further suggests the occurrence of a new promoter downstream to the breakpoints, which may be responsible for transcription-dependent R-loop formation at the *MYC* breakpoint region and, thus, fragility. Besides, our study suggests the possibility of G-quadruplex formation in two unconventional G4 motifs in Region 2.

### Bisulfite modification of chromosomal DNA from normal lymphocytes reveals long single-strandedness in Region 1 of *c-MYC* cluster II

**Figure 3:**
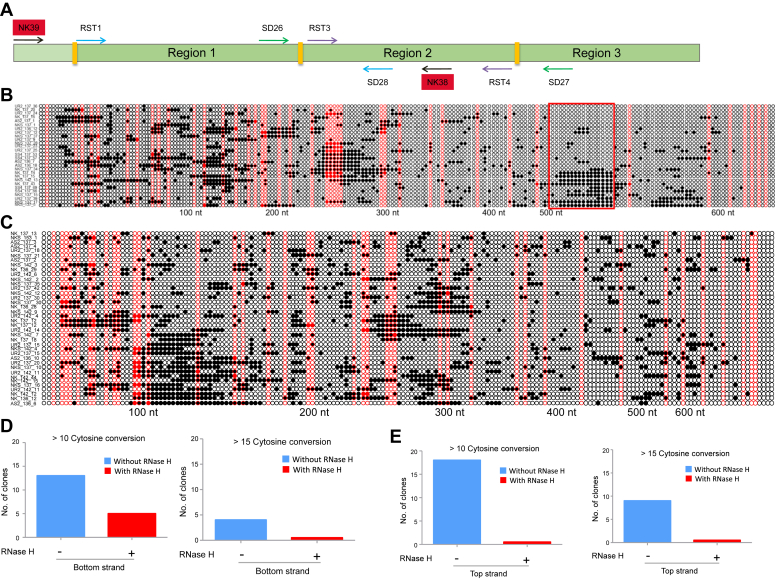
**Analysis of single-stranded regions in c-*MYC* gene on chromosomal DNA of normal lymphocytes following sodium bisulfite modification assay.***A*, schematic representation showing binding site for primers, NK39 and NK38, used for PCR amplification of extended Region 1 (688 bp), including an upstream sequence of 100 bp (*light green*) and downstream sequence from Region 2. *B*, representation of C→T conversion at the bottom strand of extended Region 1 following bisulfite modification assay. The genomic DNA of lymphocytes was used for bisulfite modification followed by PCR, TA cloning, and sequencing. A single DNA molecule is represented in each row. A total of 101 clones were sequenced of those clones with maximum conversion from the bottom strand (28) is presented. For other details refer Figure 1*C* legend. *C*, representation of bisulfite conversion of cytosine in top strand of extended Region 1 (688 bp).Thirty eight selected clones out of 60 top strands are shown. For other details refer Figure 1*C* legend. *D* and *E*, bar graph representing the number of clones with continuous cytosine conversion in the *bottom* (*D*) and *top* (*E*) strands of genomic DNA from normal lymphocytes with and without RNaseH treatment. Clones with >10 and >15 continuous cytosines conversion are plotted in both panels. When the value was zero, an arbitrary number of 0.1 was considered for plotting the graph.

The above results showed the presence of single-stranded DNA at the *c-MYC* fragile region in Raji cells where one of the chromosomes is translocated as part of t(8:14) translocation (50) and hence the genome is heterozygous with respect to the translocated allele (50, 51). Therefore, we were interested in evaluating the single-strandedness due to non-B DNA formation in normal lymphocytes when both alleles are intact. To investigate this, we isolated the chromosomal DNA from the lymphocytes of healthy individuals (3) and subjected it to a bisulfite modification assay. A 688 bp comprising extended Region 1 of *c-MYC* gene region was PCR amplified, cloned, and sequenced. Sequence analysis of 101 clones (41 bottom and 60 top) showed the presence of a long stretch of single-strandedness covering ∼100 bp in the case of the bottom strand (Fig. 3, *A* and *B*), confirming the R-loop formation in the antisense direction in the template strand. 13 clones showed continuous conversion of more than ten accounting for a region up to a length of 90 bp in the genome. More importantly, RNase H treatment resulted in no single-strandedness in this region (Fig. 3*D*). Interestingly, a closer analysis of converted molecules in extended Region 1 including its upstream revealed three different peaks of single-stranded regions (Fig. 3*B*). One of the peaks matching with G4 motif in the bottom strand (Fig. 3*B*, approximately 500–530 nt), while the other two peaks suggest the formation of shorter R-loops in the anti-physiological direction at Region 1 as well as its upstream.

Importantly we also found robust C→T conversion in the top strand of normal lymphocytes, unlike in Raji cells (Fig. 3, *A* and *C*). Out of 60 top-strand sequences analyzed, 18 had a continuous conversion of >10 cytosines of which three had a stretch conversion of more than 20 cytosines (Fig. 3, *C* and *E*). These results indicated the presence of single-stranded regions inside the genome of normal lymphocytes, even in the top strand, and suggest the formation of R-loop in physiological orientation at Region 1 and its upstream. Further, the single-strandedness disappeared following RNase H treatment (45 clones), confirming the R-loop formation (Fig. 3*E*). These results suggest the formation of R-loop both in physiological and anti-physiological orientation in the genome of normal lymphocytes.

### DRIP-sequencing analysis reveals R-loop formation at the *c-MYC* breakpoint region

**Figure 4:**
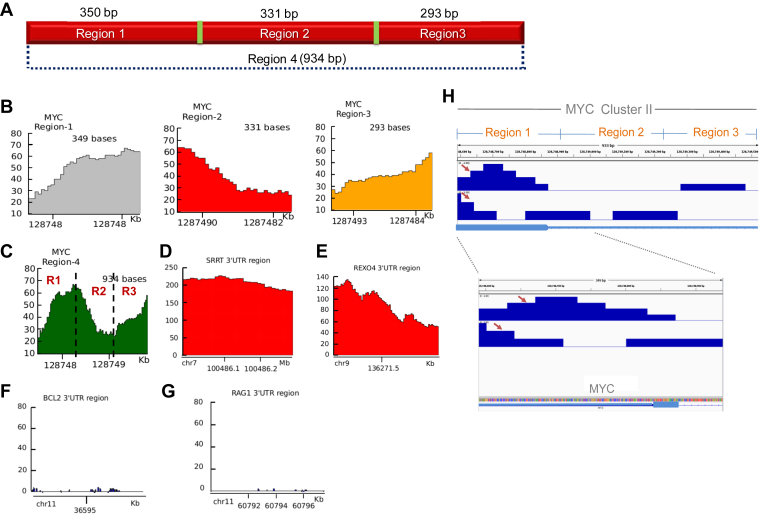
**Analysis of R-loop formation across Region 4 of the *MYC* gene based on ChIP sequencing studies.***A*, schematic representation of the 934 bp breakpoint region (Region 4), which is divided into Region 1, Region 2, and Region 3. *B*, evaluation of R-loop antibody (S9.6) binding sites in *MYC* breakpoint region based on analysis of DRIP sequencing data (52). The plots presented depict the extent of antibody binding to Regions 1, 2, and 3, with a stronger DRIP-seq signal at Region 1, suggesting a higher preference for R-loop formation at Region 1. *C*, the plot depicts the presence of DRIP-seq signals at Region 4 of the *MYC* gene. *D* and *E*, analysis of 2 positive control regions based on ChIP-sequencing. The regions shown are 3′ UTR regions from SRRT (*D*) and REXO4 (*E*) genes, which show a high enrichment of R-loop formation. *F* and *G*, representative negative controls, 3′UTR regions from BCL2 (*F*) and a RAG1 (*G*) region showing insignificant R-loop antibody binding. In panels, *B*–*G*, the x-axis represents the chromosomal location, and the y-axis represents the peak enrichment. *H*, DRIP-Seq depicting the R-loop antibody binding in Cluster II of *MYC*. This reveals the strand specificity involved in the R-loop formation. *Red arrow* represents the binding in Region 1 when two samples were analyzed. No peaks were observed in the Regions 2 and 3.

In order to further investigate R-loop formation at the *c-MYC* breakpoint region within the genome, we performed a DRIP-sequencing analysis. For this, the processed wiggle files (file that allows visualization of continuous genomic data) from the human DRIP-seq experiment, GSM1720617 conducted on the embryonic carcinoma NT2 cells using R-loop antibody (52) were downloaded from the NCBI-GEO database. The sequence analysis showed the presence of a stronger DRIP-seq signal at Region 1 as compared to Regions 2 and 3, suggesting a higher preference for R-loop formation at Region 1 (Fig. 4, *A* and *B*). Interestingly, R-loop antibody binding, which was detected as DRIP-seq signals, followed an interesting pattern when the whole breakpoint region (Region 4) was analyzed (Fig. 4*C*). Results suggest that R-loop antibody footprint covers a portion of Region 1 and the start of Region 2 (Fig. 4*C*). 3′ UTR regions from *SRRT* and *REXO* served as a positive control for the analysis in which significant enrichment of R-loop formation was observed (Fig. 4, *D* and *E*). In contrast, no detectable signal was observed when 3′ UTR regions from *BCL2* and *RAG1* were analyzed (Fig. 4, *F* and *G*), in which R-loop formation was not reported and served as a negative control.

Upon DRIPc-sequence analysis of the sequences of *MYC* breakpoint region for two samples, we observed binding of R-loop antibody in Region 1 in an anti-physiological direction, while the binding was not observed for Region 2 and Region 3 (Fig. 4*H*). Therefore, bisulfite modification assay in conjunction with DRIPc-sequencing studies suggests the formation of R-loop at extended Region 1 of the *c-MYC* breakpoint region within the human genome.

### Two intramolecular G-quadruplexes are formed on the non-template strand (top strand) of the *c-MYC* breakpoint region

**Figure 5:**
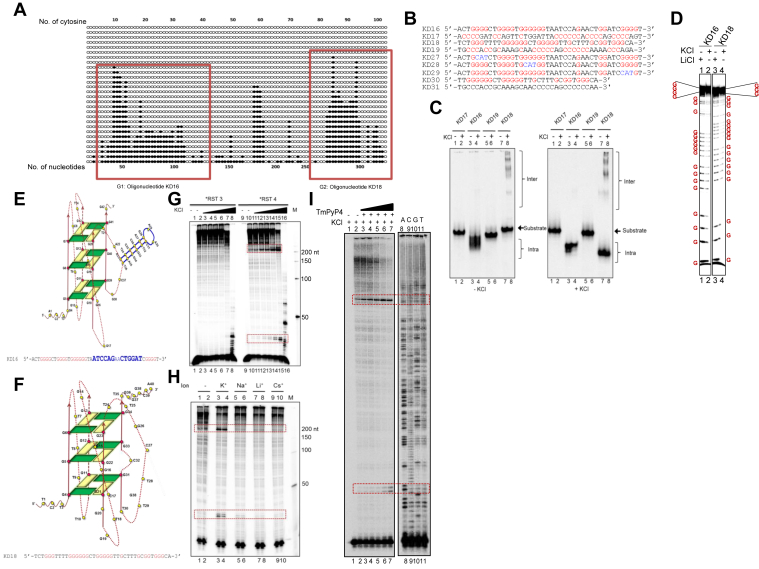
**Evaluation of G-quadruplex DNA structure formation at c-*MYC* breakpoint region.***A*, cytosine conversions at an individual molecule level following bisulfite sequencing at Region 2, when present on a plasmid (pKD2). *Dark circles* indicate the conversion of cytosine to uracil, while *white circles* indicate no conversion. *Red-boxed* regions indicate two conversion peaks spanning the sequences of oligomers, KD16 and KD18. *B*, the G-rich strands, KD16 and KD18, and their complementary C-rich strands, KD17 and KD19, respectively, along with the mutants of KD16 (KD27, KD28, and KD29) and KD18 (KD30 and KD31) are shown. *C*, KD16, KD17, KD18, and KD19 were incubated in the presence of KCl (100 mM) and resolved in the absence (*left panel*) or presence (*right panel*) of KCl (100 mM), in the gel and running buffer. The substrate, intramolecular (Intra G), and intermolecular (Inter G) quadruplex structures are indicated. *D*, DMS protection assay on KD16 and KD18 in the presence of 100 mM LiCl and 100 mM KCl. Individual guanines are marked in *red*. *E* and *F*, 2-D model representing KD16 (*E*) and KD18 (*F*) intramolecular parallel G-quadruplexes. *G*, primer extension assay was carried out on pKD2 with increasing concentrations of KCl (3, 12, 20, 50, 100, 200 mM) using 5′ labeled primers, ∗RST3 (*left panel*) and ∗RST4 (*right panel*), and the reaction products were then resolved on 8% denaturing PAGE. The pauses obtained are indicated with *red boxes*. *H*, Primer extension assay on plasmid pKD2 using oligomer ∗RST4 in the presence of 100 mM KCl, NaCl, LiCl, or CsCl. *I*, primer extension assay on pKD2 following treatment with increasing concentration of TMPyP4 (100, 250, 500 nM, 1, 2 μM) using 5′ labeled primer, ∗RST4. A sequencing ladder generated by the dideoxy sequencing method using ∗RST4 (Lanes 8, 9, 10, 11) was loaded simultaneously for better appreciation of the nucleotide position corresponding to the pauses. For other details, refer panel *G* legend.

Bisulpfite modification assay on plasmid covering Region 2 (pKD2), revealed C→T conversion on the bottom strand, independent of transcription (Figs. 5*A* and S4, *A* and *B*). Interestingly, we observed that the sequence complementary to the converted region at the bottom strand possessed two long stretches of guanines on the non-template top strand. Although the sequences present were not conventional G-quadruplex forming motifs, we investigated the potential of these two independent regions to fold into G-quadruplexes.

To test the hypothesis, oligomers spanning the two regions were designed, gel purified, and resuspended in TE (pH 8.0), and circular dichroism (CD) analysis was performed at 37 °C in the presence and absence of KCl, which is known to stabilize G-quadruplex (Fig. S5). Since G-quadruplexes are known to form only upon unwinding of DNA during physiological processes such as transcription or replication, we used ssDNA for G-quadruplex structure analysis. Interestingly, we observed that G-rich oligomers spanning regions 1 (G1) and 2 (G2), namely, KD16 and KD18, respectively, followed a spectral pattern of parallel G-quadruplex, which was stabilized in the presence of K^+^ (Fig. S5). In contrast, C-rich complementary strands, KD17 and KD19 showed spectra characteristic of a single-stranded DNA, irrespective of the presence of KCl (Fig. S5*A*). Importantly, both KD16 and KD18 do not follow the empirical formula of the G-quadruplex forming sequence, which is believed to be G≥3X G≥3XG≥3X G ≥ 3, where the loop length (X) ranges from one to seven. KD16 follows the new definition of the G-quadruplex forming sequence reported recently, where Phan’s group showed that larger loop lengths could be accommodated by folding into duplex hairpins (53, 54). The structure was stable at temperatures as high as 55 °C in both KD16 and KD18; however, further heating to 90 °C resulted in denaturation of the structure (Fig. S5*B*). Importantly, we could recreate the structure upon gradual cooling of the sample to 25 °C in the presence of KCl (Fig. S5*B*, right panel).

EMSA studies showed that the majority of KD16 and KD18 can fold into intramolecular G-quadruplex structures in the presence of KCl (100 mM) (Fig. 5, *B* and *C*). A series of mutants (KD27, KD28, and KD29) were generated for both KD16 and KD18 to delineate the relevance of guanine stretches in G-quadruplex formation (Fig. 5*B*). Further studies revealed that upon mutation of different G-stretches in KD16, intermolecular G-quadruplex formation was observed in all three mutants (KD27, KD28, and KD29), independent of KCl (Figs. 5*C* and S5*C*). Further, 5′ truncated mutants (KD30) were generated for KD18, and EMSA analysis showed that an intramolecular G-quadruplex was formed in the presence of KCl, which was also consistent with CD analysis (Fig. S5*C*, right panels). Besides, 3′ truncation of KD16 (KD45) showed that the first three stretches of guanines were sufficient for the intramolecular structure formation in parallel orientation, based on CD and EMSA analysis (Fig. S5, *D* and *E*).

In order to evaluate the precise base pairing during G-quadruplex formation on KD16 and KD18, a DMS protection assay was performed in the presence of KCl (100 mM) and LiCl (100 mM). Lithium is a monovalent cation that is known to not support the formation of G-quadruplexes. Results showed the protection of different guanines of KD16 and KD18 in the presence of KCl, while such sensitivity was reduced in the presence of LiCl, implying abrogation of G-quadruplex formation (Fig. 5*D*). Based on the results obtained from various assays described above, 2-D models were built for both KD16 and KD18. One of the possible conformations of KD16 and KD18 are depicted. Both KD16 and KD18 fold into parallel intramolecular G-quadruplex. KD16 structure involves a long loop that can fold into a duplex hairpin, as indicated in blue (Fig. 5*E*). In contrast, KD18 may harbor a bulge to accommodate the GNG motifs when it folds (Fig. 5*F*).

### Formation of G-quadruplex structures on plasmid DNA blocks DNA replication

In order to investigate whether the G-quadruplex structures are formed when the sequence motif is present on a plasmid, pKD2 was used for primer extension studies in the presence of increasing concentrations of KCl (Fig. 5*G*). For this, supercoiled DNA was purified using Cesium chloride-ethidium bromide density gradient centrifugation and used for the study. While no pauses were obtained upon primer extension on the bottom C strand using RST3 primer (template with respect to transcription reactions), two distinct pauses, corresponding to the regions G1 and G2, were observed when primer extension was carried out on the non-template (top) strand using primer RST4 (Fig. 5*G*). The formation of a G-quadruplex DNA structure on a template DNA will result in the arrest of nascent strand synthesis during DNA replication culminating in observed pause as described before (55). The intensity of the pause sites increased in a KCl concentration-dependent manner, further supporting the hypothesis (Fig. 5*G*). Further, the primer extension experiments were performed in the presence of various monovalent cations such as Na^+^, Cs^+,^ and Li^+^ (100 mM) that are not known to support G-quadruplex structure formation along with KCl (100 mM) in a parallel reaction. Na^+^ and K^+^ are abundant inside the cell, and a cellular equivalent concentration range (100 mM) was chosen for this experiment. The same concentration was used for Cs^+^ and Li^+^. K^+^ was the only cation to stabilize the two G-quadruplex structures within this region (Fig. 5*H*).

We also used a well-known G-quadruplex stabilizing agent, TMPyP4, in the study (56, 57). pKD2 was subjected to primer extension analysis in the presence of increasing concentrations of TMPyP4 (100, 250, 500 nM, 1, 2 μM). As seen in the case of KCl, TMPyP4 was able to stabilize both the G-quadruplex structures (G1 and G2) in a concentration-dependent manner, which was evident as increased intensity of the pause site obtained during primer extension (Fig. 5*I*).

In order to rule out the possibility that pause sites generated are due to potential/incomplete denaturation of the DNA and not due to G-quadruplex formation, supercoiled DNA was linearized and used for the studies. The increasing concentrations of the supercoiled or linearized pDNA (100, 200, 300, and 400 ng) were incubated in TE containing 50 mM KCl and subjected to primer extension assay. Results showed distinct pause sites in regions G1 and G2 when radiolabeled RST4 primer was used and was consistent with the above studies (Fig. S6, *A* and *B*). G-quadruplex formation on the G-rich strand of pKD2 can result in single-strandedness on the complementary region. To test this, supercoiled pKD2 was treated with increasing concentrations of P1 nuclease, and results showed a concentration-dependent increase in the sensitivity at region complementary to the G-quadruplex motif, and such sensitivity was absent in flanking DNA, except when the highest concentration of enzyme was used (data not shown).

### AID binds to *c-MYC* breakpoint region inside the cells

**Figure 6:**
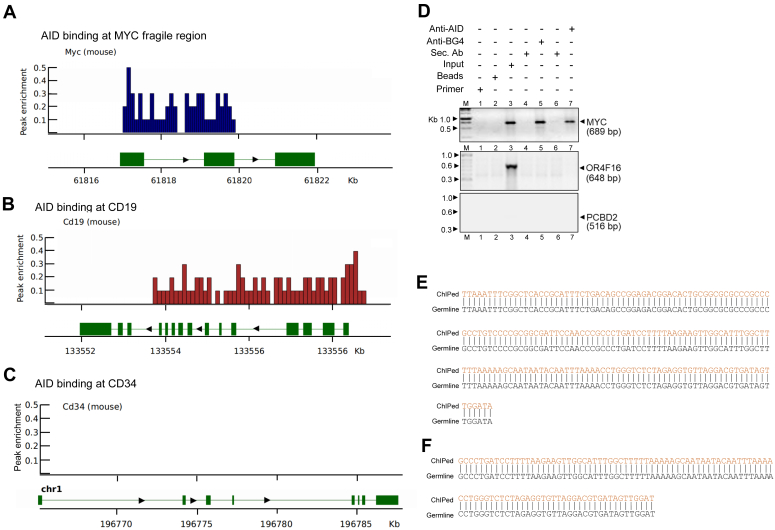
**Evaluation of AID binding to patient breakpoint regions at *MYC* following ChIP-sequencing.***A*, a ChIP-seq track is shown depicting AID binding signals present across the first two exons of the *MYC* gene. ChIP sequencing studies (60) done in mice were analyzed and presented. A genomic view of *Myc* from the mouse genome is also shown in the *lower panel*. *B* and *C*, ChIP-seq track showing AID binding efficacy across a major portion of the CD19 gene (positive control; *B*) and CD34 (negative control; *C*). A genomic view of the CD19 and CD34 genes from the mouse is also shown. In all cases, the x-axis represents the chromosomal locations, and the y-axis represents the peak enrichment. *D*, ChIP-PCR to evaluate AID binding to *MYC* breakpoint Region 1 in human cells. Representative agarose gel image showing the amplification of AID bound Region 1 and 2 of *MYC* fragile region (689 bp) following ChIP assay (*top panel*). G-quadruplex binding antibody BG4 was also used for ChIP studies. The amplification of control regions from OR4F16 (648 bp) and PCBD2 (516 bp) genes are also shown (*lower panels*). *E* and *F*, DNA sequence alignment following sequencing of BG4 (*E*) and AID (*F*) bound *MYC* region obtained after PCR and ChIP assay is presented.

Previous studies have suggested the role of AID protein in the generation of t(8;14) translocation. Using transgenic mouse models, it has been shown that AID is required for the generation of *c-MYC* translocation to the Ig switch region (23, 24, 58, 59). Considering that the formation of both R-loop and G4 DNA can generate ss DNA regions, we were interested in testing whether AID can directly bind to the *c-MYC* breakpoint region. To investigate this, firstly, we analyzed available AID-ChIP-seq experimental data from the database (60). Since human AID-ChIP-seq data was not available, we reanalyzed the data from the AID-ChIP-seq experiment conducted in activated B cells isolated from C57BL/6 mice, which was publicly available. ChIP-seq analysis showed AID binding peaks over the first two exons of the *c-Myc* gene. This was comparable to the mouse CD19 region, which served as a positive control (Fig. 6, *A* and *B*). CD34 from the mouse served as the negative control, in which no AID binding was observed (Fig. 6*C*).

To test whether AID can bind to the *c-MYC* breakpoint region in human cells, ChIP analysis was performed in Raji cells using an antibody against AID. PCR was performed to amplify the *MYC* breakpoint extended region 1 (RST1 and SD27) and two other random regions. Results showed specific PCR amplification in the case of the *c-MYC* breakpoint region, while no products were seen when the same ChIPed DNA was used in the case of control regions, OR4F16 (648 bp) and *PCBD2* (516 bp) region (Fig. 6*D*). The PCR product was sequenced to confirm the identity (Fig. 6*E*). These results suggest that AID can indeed bind to *MYC* breakpoint regions within the cells.

Since we also observed the formation of G-quadruplexes at the *c-MYC* region, we were interested in examining whether the BG4 antibody, which is specific against G-quadruplex DNA, could bind to the *c-MYC* breakpoint region (61, 62). To test this, we performed ChIP analysis using BG4 in Raji cells. PCR amplification showed that primers specific to the *MYC* breakpoint region resulted in a DNA fragment corresponding to the region of interest, while no products were observed in the case of controls, OR4F16 (648 bp) and PCBD2 (516 bp) region (Fig. 6*D*). Further, the PCR product was sequenced, and identity was confirmed (Fig. 6*F*). Thus, our results reveal that the BG4 antibody can indeed bind to the *c-MYC* breakpoint region, suggesting the existence of a G-quadruplex in the genome.

### Purified AID can bind and deaminate *c-MYC* breakpoint region

**Figure 7:**
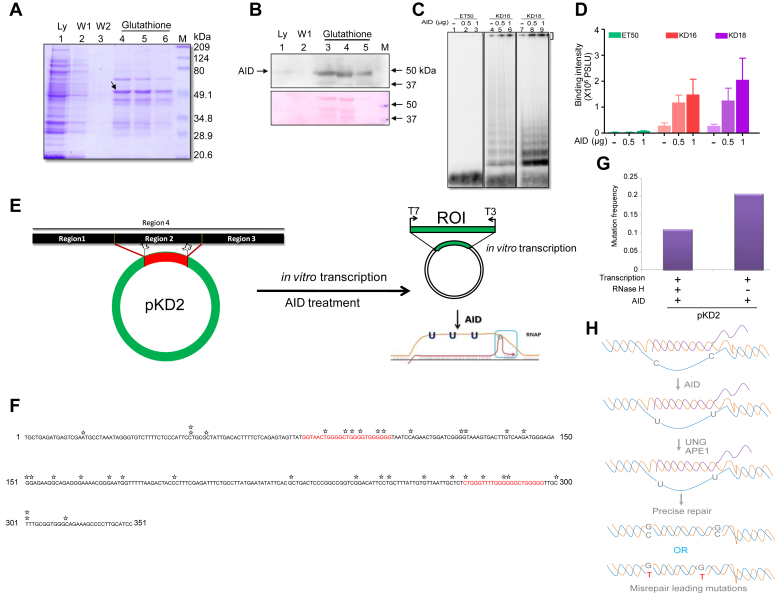
**Biochemical characterization of AID binding to a single-stranded region of *MYC*.***A*, SDS gel profile showing the purification of AID protein. A band corresponding to AID is marked with an arrow. Lane 1 is the lysate; lanes 2 and 3 represent the wash; lanes 4 to 6 represent the different elution of AID in the presence of reduced Glutathione. “M” is the marker. *B*, Western blotting to confirm the presence of AID protein after purification. Lanes 3 to 5 represent the different elution of AID. For other details, refer to panel *A* legend. *C*, native EMSA showing the binding of AID to DNA spanning *MYC* fragile regions (KD16 and KD 18). The binding of AID to the substrate is bracketed. ET50 served as a negative control. *D*, bar graph showing quantitation of AID (0.5 and 1 μg) binding to the *c-MYC* fragile region compared to control DNA (ET50). The data is plotted with error bars as mean ± SEM. *E*, schematic representation of *in vitro* transcription followed by AID treatment on pKD2 plasmid and analysis of AID-induced mutations. *F*, sequence plot showing the mutations and their position in Region 2 (denoted by ∗) of the *c-MYC* gene after transcription and treatment with AID. The sequence in red indicates G4 motifs. *G*, bar graph showing mutation frequency on transcribed pKD2 in the presence and absence of RNase H after treatment with AID protein. For studying the mutation frequency, 19 and 54 clones were analyzed for transcribed plasmids with RNase H and without RNase H, respectively. *H*, schematic representation explaining the possible mechanism of action of AID leading to imprecise repair and mutations. UNG generates an abasic site, which is then cleaved by APE1, generating single-stranded breaks. Misrepair of these breaks could lead to mutations.

To further investigate the role of AID in binding to the fragile region and its deamination, AID protein was overexpressed and purified from *E. coli* BL21 (Fig. 7*A*). The identity of purified AID was confirmed through western blotting (Fig. 7*B*). EMSA studies showed that purified AID can bind to oligomeric DNA containing G-quadruplexes in a concentration-dependent manner, although the bound fraction remained in the well itself (Fig. 7, *C* and *D*). Further, we were interested in testing whether AID can deaminate the cytosines in the *MYC* breakpoint region. To do this, purified AID was incubated with pKD2 following *in vitro* transcription in the presence and absence of RNase H. Reaction products were purified and transformed into *E. coli* Ung-strain of Bacteria. Plasmid DNA was purified and sequenced (Fig. 7*E*). Deamination of cytosine when present on a single-stranded DNA by AID can result in uracil, and the C→U conversion can be read as C→T (or G→A depending on the strand sequenced) upon replication of DNA inside bacteria followed by sequencing. Aberrant repair through base excision repair can result in other mutations as well (Fig. 7, *E* and *H*). The sequencing results of each clone were analyzed and mutations were plotted to the *MYC* breakpoint region (Fig. 7*F*). Results showed several point mutations following treatment with AID. Some of the mutations were also present at or near the G-quadruplex forming motif (Fig. 7*F*). Further, mutation frequency analysis suggests that there was a 50% reduction in mutations upon RNase H treatment (Fig. 7*G*). This also indicates that while 50% of the mutation observed could be due to R-loop formation, the other half could be contributed by G-quadruplexes. Thus, results from biochemical experiments suggest that AID can indeed deaminate cytosine present at the single-stranded region due to non-B DNA structures at the *c-MYC* breakpoint region (Fig. 7*H*).

### Overexpression of AID leads to mutations in the *c-MYC* breakpoint region in Raji cells

**Figure 8:**
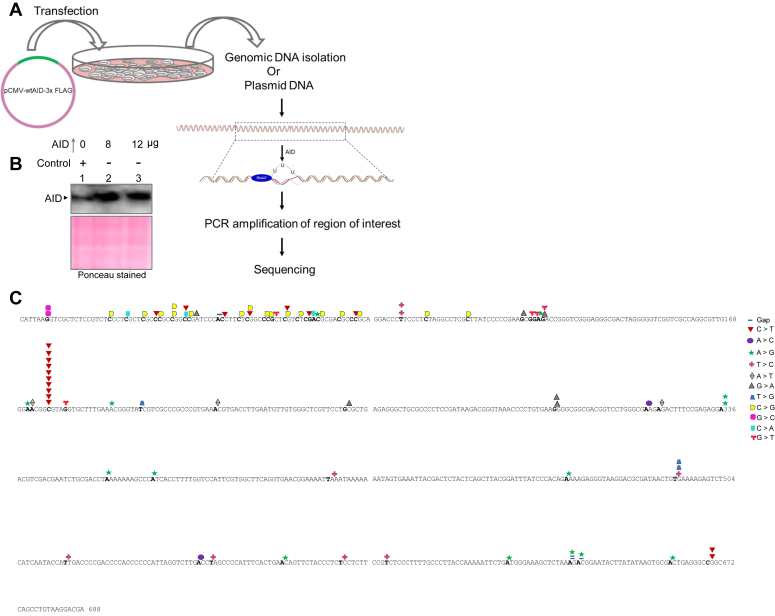
**Evaluation of mutations in Raji cells following overexpression of AID.***A*, experimental strategy used for the analysis of AID-induced mutations following overexpression of AID in Raji cells. AID overexpression vector, pCMV-wtAID-3x FLAG, was transfected into Raji cells (48 h), and genomic DNA was harvested and used for PCR amplification, followed by cloning and sequencing. A fraction of the transfected cells was used for the preparation of cell lysate for western blotting. Genomic DNA was used to amplify the region of interest, followed by sequencing, and AID-induced mutations were analyzed. *B*, Western blot analysis following ectopic expression of AID in Raji cells. The PEI alone served as the no-transfection control (Lane 1). Ponceau stained blot served as the loading control. *C*, sequence plot showing cumulative nucleotide conversions in the *MYC* fragile region (extended Region 1) upon overexpression of AID in Raji cells. A total of 100 clones were sequenced from three different batches of AID overexpressed cells, and mutations caused by the overexpression of AID were analyzed and plotted. Different symbols represent different types of point mutations.

Previous results showed that purified AID protein can bind to the *c-MYC* breakpoint region and deaminate the cytosines on plasmid DNA. Further, to investigate that AID can deaminate the cytosines at the *MYC* breakpoint region inside the cells, Raji cells were transfected with the AID expressing vector, pCMV-wtAID-3x FLAG, to ectopically express AID inside cells (Fig. 8*A*). The overexpression was confirmed by immunoblotting (Fig. 8*B*). Parallelly, genomic DNA was isolated from AID overexpressed samples, and extended Region 1 was PCR amplified, cloned, sequenced, and analyzed for AID-induced mutations. AID can deaminate the cytosines present on a single-stranded DNA and the C→U conversion can be read as C→T (or G→A depending on the strand sequenced) upon replication of DNA. Besides, aberrant repair of deaminated cytosine through base excision repair inside the cells may result in other mutations as well. Sequencing results (100 clones) showed distinct nucleotide conversions upon AID overexpression (Fig. 8*C*). We observed different nucleotide conversions, including C→T and G→A, and were AID dependent with a 0.15% mutation frequency compared to random regions (<0.002) upon sequencing. Thus, our results reveal that overexpression of AID within the cells leads to increased mutation frequency at c-*MYC* fragile regions.

### Anti-γH2AX binds to the DSBs generated at breakpoint regions of *MYC* inside cells

**Figure 9:**
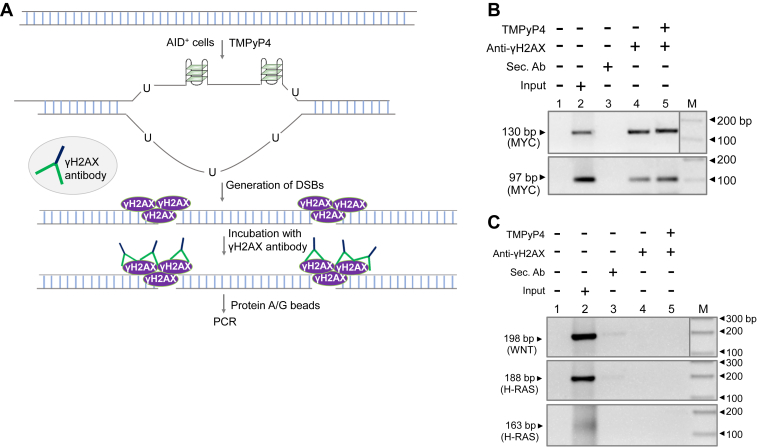
**Evaluation of formation of DNA double-strand breaks at c-*MYC* fragile region.***A*, experimental strategy used for chromatin immunoprecipitation (ChIP) assay to detect the DSBs at *MYC* region. Cells were treated with G-quadruplex stabilizing agent, TMPyP4 (5 μM). The cytosine present in the single-stranded DNA, complementary to G4 DNA, can be converted into uracil by the action of endogenous AID inside the cells, leading to the generation of SSBs and DSBs. γH2AX protein is recruited to DSBs when present inside the cells, which can be then pulled down using γH2AX antibody followed by the use of protein A/G beads and used for ChIP-PCR. *B* and *C*, ChIP-PCR to evaluate binding of γH2AX to *MYC* breakpoint region in Raji cells. The agarose gel image shows the amplification of γH2AX bound *MYC* fragile region. NK33 and NK34 primers were used to amplify the downstream of G-quadruplex forming motif 1 (130 bp; top panel), and NK35 and NK36 primers were used to amplify the downstream of G-quadruplex forming motif 2 (97 bp) from the ChIP DNA. Lane 1 is the no template control. WNT (198 bp; top panel) and H-RAS (188 bp and 163 bp; lower panels) genes served as the random controls (C).

The above results suggested that single-stranded DNA is present at c-*MYC* cluster 2 inside the cells, and AID can bind and deaminate the cytosines inside the cells. The deaminated cytosine is generally repaired by the base excision repair pathway, and failure of it can result in the generation of nick, which can be converted into staggered DSBs either because of two independent SSBs at close proximity or due to replication across the SSBs. To investigate the generation of DSBs inside the cells, we employed chromatin immunoprecipitation (ChIP) in the presence of a G-quadruplex stabilizing agent, TMPyP4 (Fig. 9*A*). ChIP using anti-γH2AX followed by PCR of the pull-down DNA was performed to amplify the downstream region of the putative G-quadruplex motif 1 and 2 of *MYC* breakpoint region. PCR of two random regions served as the control (Wnt, and H-Ras). Results showed specific PCR amplification (130 bp and 97 bp) in the case of the *c-MYC* breakpoint region near G-quadruplex forming structures, while no amplification was seen when the same ChIPed DNA was used to amplify the control regions, Wnt (198 bp; top panel) and H-Ras (188 bp and 163 bp; lower panels) (Fig. 9, *B* and *C*). These results suggest that DSBs are generated at cluster 2 of the c-*MYC* fragile region, and γH2AX can indeed bind to DSBs at c*MYC* breakpoint regions within the cells.

**Figure 10:**
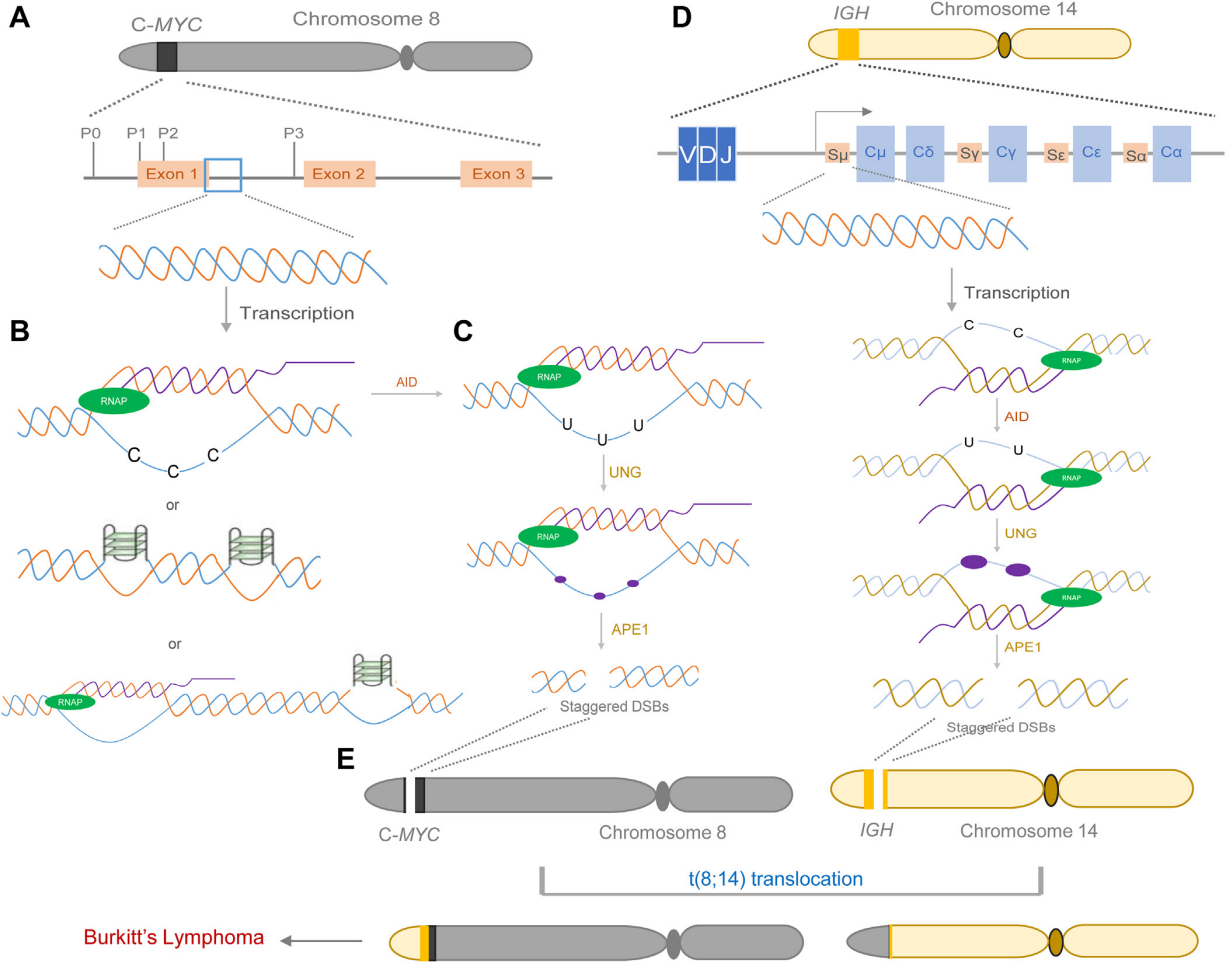
**Model explaining the mechanism of t(8;14) translocation in Burkitt’s Lymphoma.***A*, *c-MYC*, the protooncogene, present on human chromosome 8 contains four promoters (P0, P1, P2, and P3), three exons (exon 1, 2 and 3) and two introns. The *blue box* indicates the breakpoint region (934 bp) reported during t(8;14) translocation covering the portion of exon 1 and intron 1. *B*, our results showed the formation of the R-loop in an anti-physiological direction following *c-MYC* transcription and the formation of unconventional G quadruplexes on the *c-MYC* non-template strand in a mutually exclusive manner. Short R-loops are formed in overlapping regions in anti-physiological direction or/and G-quadruplex form. *C*, AID can act on the single-stranded regions of these structures converting cytosine to uracil followed by BER. UNG generates an abasic site (indicated by a *purple oval*) that is then cleaved by APE1 generating single-stranded breaks in some instances. Aberrant repair of these breaks can lead to mutations. Alternatively, concomitant breaks in the complementary strand could result in staggered DSBs. *D*, on the *IGH* locus of chromosome 14, the switch region (S*μ* region) can fold into an R-loop structure following transcription. AID action at ssDNA leads to C→U conversion, which finally leads to SSBs and DSBs, as described above. *E*, the DSBs generated on *c-MYC* on chromosome 8 and *IGH* on chromosome 14 may finally lead to t(8;14) translocation when chromosomes 8 and 14 are present in proximal regions of the nucleus, which can culminate in Burkitt’s lymphoma.

In summary, multiple lines of evidence suggest that the R-loop is formed in anti-physiological orientation at Region 1 and spans to the start of Region 2. As suggested by DRIP sequencing data and gel shift studies, R-loop may also be seen at Region 1 and 2 in an anti-physiological direction. Bisulfite sequencing, ChIP studies using BG4 antibody, and biochemical studies suggest the formation of two independent G-quadruplexes, particularly at Region 2. The formation of any of these structures will result in single-strandedness, which in turn can become a target for the AID protein. AID protein will deaminate the cytosine, which can be further processed by UNG and APE1 protein resulting in SSBs, which later can get converted as double-strand breaks, which could result in t(8;14) translocation (Fig. 10).

## Discussion

In this study, we have explored the mechanisms of *c-MYC* fragility during t(8;14) translocation with respect to breakpoint Cluster II. Considering that sporadic BL breakpoints majorly span across the exon 1-intron 1 of *c-MYC,* and AID, a cytidine deaminase that works on single-stranded DNA and is suggested to be responsible for t(8;14) translocation, a DNA structural basis for fragility was explored. Previous studies have shown unpaired loop regions on a plasmid-based system in the c-*MYC* fragile region using electron microscopy, suggesting the formation of an RNA-DNA hybrid (63, 64). Although the formation of H-DNA was suggested at the promoter region of c-*MYC* (Cluster I) as the cause for fragility (65), the mechanism of fragility at most of the breakpoints spanning exon 1- intron 1 region remained unclear.

### R-loop formation at *c-MYC* breakpoint region

Although Cluster II of the *c-MYC* breakpoint region showed no sequence motifs that support the conventional non-B DNA structure formation, gel mobility assays suggested the formation of RNA-DNA hybrid when transcription was performed in both physiological as well as anti-physiological orientation. It has been previously shown that transcriptionally active class switch regions at the IgH locus form RNA-DNA hybrids (34, 45). Interestingly, we found R-loop formation when transcription was performed in anti-physiological orientation at Region 4, which includes both Regions 1 and 3. RNase H digestion data confirmed these results.

The bisulfite modification assay on the genome of normal lymphocytes revealed the R-loop formation in both physiological and antiphysiological orientation and was sensitive to RNaseH. Interestingly, we could not find any evidence for the occurrence of R-loop formation at physiological orientation in the genome of Raji cells based on bisulfite modification assay. Promoter prediction server analysis suggested the presence of potential promoters in anti-physiological orientation at the *c-MYC* breakpoint region, explaining the possibility of R-loop formation in the anti-physiological orientation of extended Region 1 observed in the case of both normal lymphocytes and Raji cells (Fig. S7) (66). This is not unexpected as it is suggested that up to 70% of coding genes do possess transcription in an anti-physiological direction (67). Besides, analysis of DRIP sequencing data suggested the presence of R-loop formation in Region 1 and at the start of Region 2 (Fig. 4). However, the length of the observed R-loop was short and was found to be ranging from 50 to 100 nt. The observed length of the R-loop in the case of *c-MYC* Region 1 was comparable to the RNA length previously described for switch regions (34).

Previous reports have suggested that GC skews favor R-loop formation *in vitro* as well as *in vivo* (68, 69). Our data suggest that the R-loop formation in physiological orientation is GC skew dependent, and hence, Region 2 showed maximum gel mobility shift when T7 RNA polymerase was used when compared to Region 1 and Region 3, which were devoid of GC-rich sequence (Fig. 1*C*). In contrast, a gel mobility shift was observed when T3 RNA polymerase was used for transcription in the antisense direction in the case of Regions 1, 2, 3, and 4. Consistent with this, bisulfite modification revealed the presence of single-strandedness in both top and bottom strands suggesting transcription in both physiological as well as anti-physiological transcription. However, bisulfite modification in Raji cells showed the presence of transcription only in anti-physiological orientation and it might be due to the active state of anti-physiological transcription in Raji cells. Recently, *cis*-NATs (*Cis*-natural antisense transcripts are long noncoding RNAs transcribed from the overlapping coding and noncoding genes on the sense strand) are shown to be present at the overlapping exon1/intron1, 3′UTR and 3′ distal region of the *c-MYC* gene (66). Further, they have shown that *cis*-NATs in the 3′ distal region are involved in tight regulation of c-*MYC* gene in the prostate cancer cells (66). We anticipate that in since Raji cells are cancerous cells, transcription state and regulation can be different and could explain R-loop formation on the bottom strand.

One of the early studies has shown antisense transcription of c-*MYC* gene in HeLa, Burkitt lymphoma BL-60 t(8;22) cells, and diploid fibroblasts and mapped with the first exon of the c-*MYC* gene *via* primer extension technique (70). However, the direction of the transcription was not reported. Our results suggest the occurrence of R-loop formation and transcription in an anti-physiological direction at the junction of exon 1 and intron 1 could be related to the previously reported antisense transcripts of c-*MYC* gene.

### Unusual G-quadruplexes within *c-MYC* breakpoint region

*c-MYC* Pu27, a G-quadruplex forming motif, is one of the well-characterized examples of regulatory promoter G-quadruplexes (71, 72). In our study, we have characterized two unusual G-quadruplexes at Region 2 of *c-MYC* fragile region. Bisulfite modification assay showed two stretches of single-strandedness on the template (bottom) strand, (G1 and G2) within Region 2, both in the human genome and on a plasmid DNA (Figure 2, Figure 3 and 5). Further, ChIP studies using BG4 antibody showed that G-quadruplex specific antibodies could specifically bind to the *c-MYC* region, suggesting the formation of G4 structure within the genome. This was intriguing because no structure was predicted by Non-B DB. Upon analysis of the oligonucleotides spanning the region through CD and EMSA studies, we observed robust parallel intramolecular G-quadruplex structure formation in the presence of KCl. These regions were able to fold into G-quadruplex in a plasmid context as well, which blocked replication. We characterized the structures further, showing precisely the involvement of guanines during the structure formation through DMS protection assay. Since these G-quadruplex structures utilized sequences that are deviations of consensus G-quadruplex motifs, non-B DNA predicting tools were unable to score them. Detailed analysis of the sequences showed that these G-quadruplexes involve duplex hairpin and GNG motifs during structure formation. This result is in coherence with the studies by Phan and other groups, which showed that G-quadruplex could harbor long loops only if they form duplex hairpin (53, 73). Besides, we also showed that both the G-quadruplexes were highly thermostable and were able to fold back upon renaturation.

We and others have shown that G-quadruplex structures can result in genomic instability owing to the generation of single-strandedness (2, 4, 7, 19, 74). One of the recent studies suggests that stabilization of G-quadruplexes can enhance the formation of R-loop and hence induce DNA damage and genomic instability (75). These structures are more susceptible to several cellular enzymes like RAG and AID (59). Unlike R-loops, G-quadruplexes may form in a transcription-dependent or independent manner and can cause replication/transcription block leading to DSBs and genomic instability, as could be the case in AID-independent t(8;14) translocations.

### AID protein can bind and deaminate the single-stranded region present at the *c-MYC* breakpoint region

Several studies have suggested AID as the major contributor for *c-MYC/IGH* translocation (23, 24, 26, 58, 59, 67, 76, 77). Since AID activity is preferred on single-stranded DNA templates, R-loops can be potent targets for AID. The presence of translocation breakpoints and AID motifs at *c-MYC* Cluster II reinstates AID to be a key player in chromosomal rearrangements (Fig. S1, *B* and *C*).

ChIP-DRIP sequencing data analysis revealed that AID protein could indeed bind to the *c-MYC* gene in mice, unlike random DNA control regions (60). Further ChIP studies using human cells revealed that AID could bind to the *c-MYC* breakpoint region. Besides, biochemical assays using purified AID protein demonstrated that AID could bind to G4 DNA structures present in oligomeric DNA. Importantly, we also found that purified AID, upon incubation with transcribed plasmid DNA in the presence and absence of RNase H induce mutations following transformation into bacteria. Importantly, the frequency of mutation decreases by 50% upon RNase H treatment before AID treatment suggesting that AID could target cytosines present at single-stranded DNA generated both due to G4 DNA and R-loop since plasmid selected was able to fold into R-loop as well as G4 DNA. Furthermore, overexpression of AID in Raji cells resulted in the accumulation of point mutations. These nucleotide alterations were absent in the control cells, further confirming the hypothesis that AID can indeed bind to the single-stranded region of *MYC* breakpoint and deaminate the cytosine resulting in DNA breaks. γH2AX is a molecular marker for DNA damage initiation and is an early cellular response to the generation of DSBs. ChIP studies using γH2AX antibody showed that DSBs are present at the *c-MYC* fragile region and were dependent on G4 DNA formation. Generation of DSBs could result in t(8:14) translocation (Fig. 10).

### Mechanism of t(8;14) translocation

Based on our results, two models are proposed to explain the fragility at exon 1 intron 2 junction of the *c-MYC* breakpoint region (Fig. 10). Our results provide evidence for the formation of R-loop in the anti-physiological direction in Region 1 and at the start of Region 2, and two unusual G-quadruplex structures at Region 2 as independent events. While the R-loop is transcription dependent, G-quadruplex may form when DNA unwinds during replication or transcription or simply due to negative supercoiling. Nevertheless, it is also possible that both structures may coexist due to negative supercoiling on the template strand during active transcription. Considering that the formation of these structures results in unpaired DNA, such regions can become direct targets for AID.

As discussed above, in the context of t(8;14) translocation, AID could be a major player contributing to fragility. Hence, it can be envisaged that AID can act on these single-stranded regions converting the cytosines to uracil, which is then expected to undergo base excision repair (BER). Uracil is recognized by Uracil N Glycosylase (UNG), generating an abasic site, followed by an AP endonuclease (APE) cleavage. The nicks generated due to failure of BER can be converted into staggered DSBs by concomitant breaks in proximity or during replication. Finally, end processing can result in DSB generation. CSR being an associated event can result in swapping of chromosomal arms leading to *c-MYC/IGH* translocation (Fig. 10).

Although both G-quadruplex and R-loops are implicated in genomic instability, the occurrence of DNA lesions due to these structures is limited by a plethora of resolvases inside the cell. Rec Q, WRN, and BLM helicases are well known to resolve G-quadruplex structures, whereas, Pif1, Senataxin, RNase H1/2 resolve R-loops (27, 78). Thus, the generation of chromosomal translocations will be a rare event. Previous studies from our lab have shown that chromosomal translocations can be harbored by asymptomatic individuals, implying the importance of secondary hits for oncogenesis (2, 7, 19).

In summary, our study delineates one of the mechanisms of fragility at the non-promoter region of c-*MYC* cluster II. We provide several pieces of evidence to suggest the formation of the R-loop and G4 DNA at fragile region Cluster II. Further, we show that the cytosine present at single-stranded region can become the target for AID deamination, which upon processing by DNA repair machinery could lead to DNA breaks culminating into translocation.

## Experimental procedures

### Enzymes, chemicals, and reagents

Chemicals and reagents used in the study were purchased from Sigma Chemical Co and SRL unless indicated otherwise. DNA modifying enzymes were obtained from New England Biolabs. DNA midi preparation kit was obtained from Qiagen. Gel elution kit was obtained from Sigma-Aldrich. Wizard DNA purification system was obtained from Promega (Wisconsin, USA). Radioisotope- γ-^32^P-ATP was obtained from BRIT.

### Oligomers

Oligomers were synthesized by Sigma-Aldrich, and Juniper Life Sciences. The oligomers used in this study are listed in Table S1. The oligomers were purified using 15% denaturing PAGE as described previously (79, 80), resuspended in TE, and stored at −20 °C.

### Plasmids and bacterial strains

The plasmid expressing AID protein, pCMV-wtAID-3x FLAG was a gift from Dr Tasuku Honjo, Japan and GST-AID expression construct pGEX-5X-3p was a gift from Dr Alberto Martin, Toronto, Canada. Rosetta *BL21* (DE3) pLysS cells were purchased from Novagen.

### 5′ end-labeling of oligomers

The 5′ end-labeling of the oligomeric DNA was performed using T4 polynucleotide kinase in a buffer containing 20 mM Tris-acetate (pH 7.9), 10 mM magnesium acetate, 50 mM potassium acetate, 1 mM DTT and [γ-^32^P] ATP at 37 °C for 1 h, as described previously (7, 9). The radiolabeled substrates were purified using Sephadex G-25 column and stored at −20 °C until use.

### Mapping of human *c-MYC/IGH* breakpoints

To map the *MYC/IGH* breakpoints, a portion of the *Homo sapiens* chromosome 8 genomic contig, GRCh37.p10 Primary Assembly (NCBI Reference Sequence: NT_008046.16) sequence was retrieved using NCBI human genome map viewer. The sequence was then subjected to NCBI nucleotide BLAST. The BLAST result was screened for *H. sapiens* DNA from Burkitt lymphoma/leukemia patients carrying the t(8;14) (q24;q32), with *IGH-MYC* fusion. Based on the accession numbers for all hits, individual sequences were retrieved. These sequences represent the translocated c-*MYC/IGH* junctions and contain sequences of c-*MYC* (Chromosome 8) as well as *IGH* (Chromosome 14). Further BLAST analysis of each sequence with the *H. sapiens* chromosome 8 genomic contig led to the identification of breakpoint junctions. Besides, all the sequences representing c-*MYC/IGH* patient breakpoints mentioned by Busch *et al.* (44), were retrieved and analyzed using NCBI BLAST and then plotted onto the *H. sapiens* chromosome 8 genomic contig. A total of 143 chromosomal translocation breakpoints from Burkitt’s lymphoma were mapped and presented.

### Cloning of *c-MYC* breakpoint cluster II

The *c-MYC* breakpoint cluster II (Region 4; starts 277 nt downstream of P1; a total of 934 bp) and subclusters [Region 1 (starts 277 nt downstream of P1; a total of 350 bp), Region 2 (starts 607 nt downstream of P1; a total of 331 bp) and Region 3 (starts 918 nt downstream of P1; a total of 293 bp)] were cloned in TA vector (Genei, Bangalore). It was further subcloned into the EcoRV site of pBluescript SK+. The clones containing Region 1, Region 2, Region 3, and Region 4 in forward orientation were named pKD1, pKD2, pKD3, and pKD4, respectively (Fig. 1*A*). pSCR1, a control plasmid containing a 300 bp region of *BCL2* gene cloned in pBlueScript SK+, was also used for the assays (4, 33). Large-scale plasmid preparation of all the plasmids was carried out using Qiagen plasmid Midi Kit to obtain >95% supercoiled plasmids.

### *In vitro* transcription and gel retardation assay

pKD1, pKD2, pKD3, pKD4 and pSCR1 (1 μg) were transcribed with either T7 or T3 RNA polymerases (Fermentas) in 1X transcription buffer [5× buffer: 200 mM Tris-HCl (pH 7.9 at 25 °C), 30 mM MgCl_2_, 50 mM DTT, 50 mM NaCl, 10 mM spermidine] at 37 °C for 30 min. The transcription reaction was terminated by incubating the reaction at 70 °C for 15 min. The mixture was then subjected to RNase A treatment (100, 300, and 500 ng) at 37 °C for 40 min. The transcribed plasmid was loaded on a 1% agarose (without ethidium bromide) and resolved by electrophoresis in Tris-borate-EDTA (TBE) buffer (89 mM Tris-borate, 2 mM EDTA, pH 8.4) at 25 V for 13 h. The gels were visualized using an ultraviolet illuminator after staining them with ethidium bromide (EtBr) (34, 68, 81).

### Detection of single-strandedness using bisulfite modification assay

Bisulfite modification was performed as described previously (4, 33, 34, 47). Genomic DNA isolated from Raji cells or previously isolated genomic DNA from normal lymphocytes (82) was used for the study (studies were approved by the institutional animal ethical committee (IBABIEC-01/PR05/27092023)). The genomic DNA (∼1 μg) was resuspended in 30 μl of distilled water, mixed with 12.5 μl of 20 mM hydroquinone and 457.5 μl of 2.5 M sodium bisulfite (pH 5.2), and incubated for 16 h at 37 °C in the dark. Genomic DNA from normal lymphocytes treated with RNase H for 1 h at 37 °C was also used for bisulfite modification assays.

The bisulfite-treated DNA was purified with the Wizard DNA clean-up system (Promega) according to the manufacturer’s instructions. Purified bisulfite-treated DNA was desulfonated with 0.3 M NaOH at 37 °C for 15 min. Desulfonated DNA was precipitated with 96 μl of 7.5 M ammonium acetate and 768 μl of ethanol at −20 °C for >2 h, washed with 70% ethanol, and resuspended in Tris–EDTA (TE) buffer (pH 8.0). The same was also performed in the case of plasmid DNA, where 1 μg of purified transcribed plasmid was used for bisulfite modification assay.

PCR with bisulfite-modified chromosomal DNA was carried out using a different set of primers, RST1 and SD28, SD26 and SD27, NK39 and NK38, and RST3 and RST4 were used to amplify extended Region 1 (484 bp), Region 2 overlapped with Region 1 and 3 (414 bp), extended Region 1 covering the sequence from upstream of Region 1 and overlapping sequence from Region 2 (688 bp) and Region 2, respectively. Different regions were amplified using a specific set of primers at an annealing temperature of 61 to 65 °C. PCR was carried out using the following conditions: 95 °C for 5 min (1 cycle), 95 °C for 45 s, 61 to 65 °C for 45 s, 72 °C for 45 s (34 cycles), and final extension for 5 min.

PCR using bisulfite pDNA as a template was carried out with the native primers KD13 and KD14. The PCR products were resolved on agarose gel, and the correct-sized fragment was recovered. Purified PCR products were cloned with a TA cloning kit (Genei). Plasmid DNA was isolated from each clone using the alkaline lysis method. The sequencing of the clone was carried out at Barcode BioSciences and Medauxin.

### Electrophoretic mobility shift assay

The radiolabeled oligomers were incubated either in the presence or absence of 100 mM KCl in TE buffer (pH 8.0) at 37 °C for 1 h. The reaction species were then resolved on 15% native polyacrylamide gels in the presence or absence of 100 mM KCl, both in the gel and the buffer, at 150 V at room temperature as described before (7, 19, 83). The gels were dried and exposed to a screen, and the signal was detected using phosphorImager FLA9000 (Fuji).

### Circular dichroism

The oligomers were incubated either in the presence or absence of 100 mM KCl, in TE at 37 °C for 1 h. The circular dichroism (CD) spectra were recorded at 25 °C, 55 °C, and 90 °C from 200 to 300 nm, and five cycles were accumulated for every sample, using a JASCO J-810 spectropolarimeter at a scan speed of 50 nm/min as described previously (19, 80). A separate spectrum was measured for the buffer alone for five cycles and was subtracted from all the experimental spectra. The ellipticity was calculated using the software Spectra Manager and plotted as a function of wavelength (33).

### DNA sequencing

Cycle DNA sequencing is a technique in which asymmetric PCR is used to generate an ss template for sequencing by the Sanger dideoxy-chain termination method (4, 7). Four amplification reactions were set up, each containing the same labelled oligonucleotide primer, RST3 or RST4 and different chain-terminating ddNTPs. Two cycling programs were carried out. In the first program, reaction mixtures (plasmid DNA, all four dNTPs + one ddNTP, Taq Polymerase, and labeled primers) were subjected to 25 rounds of conventional thermal cycling. After PCR, the samples were loaded in denaturing buffer onto 8% denaturing polyacrylamide gel. The gels were exposed to phosphorImager screens after drying.

### DMS protection assay

Radiolabelled oligomers were incubated in TE in the presence of either 100 mM KCl or LiCl at 37 °C for 1 h. Dimethyl sulfate (DMS) was added to the reaction mixture (1/200 dilution) and incubated for 15 min at room temperature as described before (7, 83). An equal volume of piperidine (10%) was added to each reaction which was then incubated at 90 °C for 30 min. The reaction volume was doubled using ddH_2_O and then vacuum-dried. The pellet was further washed several times and dried using a speedvac concentrator. The reaction products were resolved on a 15% denaturing polyacrylamide gel, dried, and visualized as described above.

### Primer extension assay on supercoiled plasmid DNA

Plasmid pKD2 was prepared by cesium chloride-ethidium bromide purified density gradient centrifugation and by using Qiagen DNA Midi kit (for all other experiments). The purified supercoiled plasmid was incubated in the presence of 50 mM KCl for 60 min at 37 °C. The primer extension reactions were carried out as described before (7, 55, 84) by mixing respective supercoiled DNA samples in 1X Thermo polymerase buffer [10 mM KCl, 10 mM (NH4)_2_SO_4_, 20 mM Tris-HCl (pH 8.8), 4 mM MgSO_4_ and 0.1% Triton X-100], 4 mM MgSO_4_, 200 μM deoxynucleoside triphosphates (dNTPs), 0.5 μM end-labeled oligomers, and 1 U Vent (exo-) polymerase. Linear amplification primer extensions were carried out in a PCR machine (25 cycles) under the following conditions: 95 °C for 3 min (1 cycle), 94 °C for 45 s, 58 to 64 °C for 45 s, 72 °C for 45 s, and final extension for 3 min. The annealing temperatures of the primers used were 58 °C for RST4 and 60 °C for RST3. The reactions were terminated by adding a dye containing formamide, and products were resolved on an 8% denaturing polyacrylamide gel. The gel was dried, and signals were detected using phosphorImager. Primer extension assay was carried out on pKD2 with increasing concentrations of KCl (3, 12, 20, 50, 100, 200 mM) and TMPyP4 (100, 250, 500 nM, 1, 2 μM) and also in the presence of 100 mM KCl, NaCl, LiCl, or CsCl.

### AID overexpression and purification

BL21 (DE3) *E. coli* cells were transformed with the pGEX-5X-3p expression construct and AID purification was carried out as described earlier (85). Briefly, the bacteria were grown to log-phase culture, and protein expression was induced by the addition of 1 mM IPTG followed by 16 h of incubation at 16 °C. The cells were harvested and lysed in lysis buffer (1× PBS, 1% Triton X-100, 1 mM PMSF). The lysate was loaded on a glutathione-sepharose column (GE) and the protein was eluted with 10 mM reduced glutathione. The purity of the protein was checked on SDS-PAGE and the identity of the purified protein was confirmed by immunoblotting.

### Analysis of AID-induced mutation

pKD2 (∼100 ng) was transcribed with T7 RNA polymerases (Fermentas) in 1X transcription buffer at 37 °C for 30 min as described above. The transcription reaction was terminated by incubating the reaction at 70 °C for 15 min. The mixture was then subjected to RNase A treatment at 37 °C for 40 min and control samples were treated by RNase H (37 °C for 40 min). The reaction products were purified by phenol:chloroform extraction and precipitation. 50 ng of purified AID protein was then incubated with purified DNA (1 h at 37 °C). AID-treated plasmids were then purified following deproteinization and subjected to PCR using primers RST3 and RST4. Region 2 was amplified using RST3 (forward) and RST4 (reverse) primers with an annealing temperature of 65 °C. PCR was carried out using the following conditions: 95 °C for 5 min (1 cycle), 95 °C for 45 s, 65 °C for 45 s, 72 °C for 45 s (34 cycles), and final extension for 5 min.

PCR products were resolved on an agarose gel and the correct-sized fragment was cloned into the EcoRV site of pBluescript SK+. Plasmid DNA was isolated from each clone using the alkaline lysis method; the identity of the clones was confirmed following restriction digestion analysis and sequencing. AID-induced mutations were analyzed following DNA sequencing (Medauxin, Bangalore, India). Mutation frequency was analyzed as the number of mutations per total nucleotide sequenced.

### Electrophoretic mobility shift assay using AID protein

Radiolabeled oligomers corresponding to G-quadruplex forming region derived from *MYC* breakpoint region (KD16 and KD18) and control DNA (ET50) were heat denatured in the presence of 100 mM KCl at 95 °C and allowed to renature by slow cooling. These oligomers were then incubated with increasing concentrations of AID (0.5 and 1 μg) at 4 °C for 1 h. The bound complexes were then resolved on 4% native polyacrylamide gels in the presence of 100 mM KCl (both in the gel and the buffer) at 100 V for 12 h in room temperature (7). The gels were dried and exposed to a screen, and the signals were detected using phosphorImager FLA9000 (Fuji).

### Chromatin immunoprecipitation

ChIP was performed as described with modifications (79, 86). Briefly, Raji cells were grown for 24 h at 5% CO_2_, 37 °C in an incubator. For ChIP analysis in the presence of G4 stabilizing agent, Raji cells were incubated with 5 μM of TMPyP4 for 12 h (83, 87, 88). Cells were then crosslinked with formaldehyde to a final concentration of 1%, further quenched by adding 100 μl of 1.375 M glycine per ml of culture. Cells were then pelleted down, and lysed using cell lysis buffer (5 mM PIPES, 85 mM KCl, 0.5% NP40) and then nuclei lysis buffer [50 mM Tris (pH 8), 10 mM EDTA, 1% SDS]. Lysed samples were then sonicated using Bioruptor (Diagenode) with 30 s on/45 s off pulse for 30 cycles. Sonicated chromatin was then stored at −80 °C for a minimum of 8 h. For chromatin immunoprecipitation, the chromatin was thawed in ice and centrifuged at high speed (14,000 rpm) for 15 min. The supernatant was collected, and DNA purity (A_260/280_) was measured using Nanodrop 2000, ThermoFisher. Samples were divided for input (5% chromatin), secondary control, and experimental. AID (sc-517548, Santa Cruz) or BG4 or γH2AX (BioLegend, 613401) or IgG anti-mouse IgG (Santa Cruz, sc-2025)antibodies were added to the sample and incubated for 8 to 10 h. Protein A/G-agarose beads (Santa Cruz) were added to the chromatin samples and incubated for 2 h. Samples were washed using high salt wash buffer [50 mM HEPES (pH 7.9), 500 mM NaCl, 1 mM EDTA, 0.1% SDS, 1% Triton X-100, 0.1% deoxycholate] in repeated cycles of centrifugation and then reverse cross-linked by incubating overnight at 65 °C. Finally, DNA was purified by phenol:chloroform extraction and precipitated. Three independent batches of ChIP were performed.

The regions of interest from the c*MYC* gene were amplified from the AID-ChIPed DNA using specific primers (RST 1 and RST4), while primers for OR4F16 (RBK21 and RBK22; 648 bp) and PCBD2 (RBK17 and RBK18; 516 bp) were used as negative control (Fig. 6).

ChIPed DNA using anti-γH2AX (Fig. 9) was used for amplification of c-*MYC* region using different set of primers, NK33 and NK34 (130 bp) and NK35 and NK36 (97 bp). In this case, H-Ras (SV64 and SV65; 163 bp and SV62 and SV63; 188 bp) and WNT (SV54 and SV55; 198 bp) were used as negative controls. PCR was carried out using the following conditions: 95 °C for 5 min (1 cycle), 95 °C for 45 s, 61 to 65 °C (depending on the primer sets) for 45 s, 72 °C for 45 s (34 cycles), and final extension for 5 min.

### Analysis of mutation frequency in Raji cells after AID overexpression

Raji cells were transfected with the plasmid pCMV-wtAID-3x-FLAG expressing AID. Overexpression of AID was performed using polyethylimine (PEI) method of transfection as described earlier (31, 89). In brief, ∼8 lakh Raji cells were transfected with the expression plasmid (8 and 12 μg), mixed with PEI (1:2) and OptiMEM medium (GIBCO). The PEI and plasmid mixture was then added to the cells and incubated for 48 h at 37 °C. Cells were harvested, and protein was extracted using RIPA extraction method. AID overexpression was confirmed by western blotting using anti-AID antibodies. Parallelly, genomic DNA was isolated from the same batch of cells and was used for PCR amplification of extended Region I of *MYC* using specific primers. Extended Region I was amplified using NK39 (forward) and NK38 (reverse) primers with an annealing temperature of 61 °C. PCR was carried out using the following conditions: 95 °C for 5 min (1 cycle), 95 °C for 45 s, 61 °C for 45 s, 72 °C for 45 s (34 cycles), and final extension for 5 min.

The PCR products were subsequently cloned into pDrive vector (QIAGEN) and the identity of the insert was confirmed by restriction digestion analysis followed by DNA sequencing analysis (Barcode BioSciences).

### Dataset for analysis

The datasets used to check the presence of R-loop and binding of AID to Region 4 of the *Myc* gene were downloaded from the NCBI-GEO database. To check for R-loop formation, processed wiggle files (file t∖at allows visualization of continuous genomic data) from human DRIP-seq experiment GSM1720617 using NT2 cell lines were downloaded (52). For AID binding, processed bed graph (similar to wiggle) files from mouse AID-ChIP-seq experiment were downloaded (GSM594829), resting splenic B cells from 6 to 8 weeks old wild type C57BL/6 mice were isolated using anti-CD43 microbeads, activated them in culture (using LPS and IL4) and used them for ChIP-seq analysis. A total of 57 mice were used for the study (60).

### Checking for R-loops and visualization of peaks

Locations belonging to Region 4 of the *MYC* gene, for human genome version hg19, and mouse genome version mm10 were extracted using the BEDtools suite, AWK: a featured programming language in UNIX-based operating systems and shell scripting (52). The resulting bed graph files were used to visualize and plot peaks using the Sushi package in R (scripting language). Positive control and Negative control regions were also plotted. For the positive control, 3′ UTR regions of the genes SRRT and REXO4 were used, whereas for the negative control, 3′ UTR regions of genes RAG1 and BCL2 were used.

For R-loop analysis of the *MYC* antisense strand, DRIPc-seq samples from the negative strand of the same study as DRIP-seq, GSM1720613, and GSM1720614 were used, and the peaks were visualized for Region 4 of the *MYC* gene.

### Promoter prediction

In order to check for the presence of promoter-like sequences in the anti-sense strand of Region 4, the reverse complement sequence of Region 4 was extracted and run for prediction of transcription start sites using the “Promoter prediction server”. These predicted start sites are speculated to transcribe Region 4 in the anti-physiological direction and subsequently correlate with the presence of DRIPc-seq peaks from the negative strand suggesting the formation of R-loops in the antisense strand.

### Western blotting

For checking the identity of the purified protein, ∼20 μg of lysate, wash, and three different fractions of purified protein were resolved on 8% SDS-PAGE. Western blotting was done as described earlier (89, 90, 91). Upon gel electrophoresis, resolved proteins were transferred to the PVDF membrane (Millipore). The membrane was blocked with 5% skimmed milk powder for 1 h at room temperature (RT) and probed with anti-AID antibody (1:500) (sc-517548, Santa Cruz) overnight at 4 °C, followed by incubation with biotinylated anti-goat secondary antibody (Santa Cruz; 1:10,000) for 2 h at RT. The blots were washed with PBST and then incubated with streptavidin-HRP (1:10,000) (Sigma) at RT for 30 min. The blots were developed using a chemiluminescent solution (ImmobilonTM western; Millipore) and scanned by a chemiluminescence system (LAS 3000, Fuji).

### Actinomycin D treatment and isolation of chromosomal DNA

Raji cells (4 × 10^6^) were incubated with 0.5 μM actinomycin D (Sigma) for 12 h at 37 °C. Cells were harvested and chromosomal DNA was isolated (three different batches) from Raji cells, as described before (4, 34). Briefly, cells were lysed with buffer containing 10 mM Tris and 1 mM TE buffer (pH 8.0) with 0.5% sodium dodecyl sulfate and incubated with Proteinase K overnight at 37 °C. Genomic DNA was purified by phenol:chloroform extraction and precipitated using ethanol. Air-dried genomic DNA was resuspended in TE buffer and used for the bisulfite treatment.

### Plasmid isolation and EtBr-CsCl purification

*E.coli* transformed with each plasmid were grown at 37 °C for 16 to 18 h. Plasmid DNA of interest was isolated from *E.coli* by standard alkaline lysis method and purified by Caesium chloride (CsCl)-ethidium bromide (EtBr) density gradient centrifugation (92, 93). Briefly, CsCl solution was prepared by adding 5.1 g CsCl in 4.0 ml TE, following which 5 mg of plasmid DNA and 0.7 mg EtBr were added. This mixture was vortexed and transferred into quick seal tubes and centrifuged (Beckman Coulter) at 72,000 rpm, 20 °C for 12 h. Plasmid DNA was extracted by removing EtBr by butanol extraction and then DNA was precipitated using isopropanol. Plasmid DNA was washed with 70% ethanol and air-dried pellet was resuspended in TE (pH 8.0).

## Data availability

All data supporting the findings of this study are available in the manuscript and in the Supplementary materials.

## Supporting information

This article contains supporting information.

## Conflict of interest

The authors declare that they have no conflicts of interest with the content of this article.
