# Supplementary material for: Evaluation of potential role of R-loop and G-quadruplex DNA in the fragility of c-MYC during chromosomal translocation associated with Burkitt’s lymphoma
Source: J Biol Chem. 2023 Nov 4;299(12):105431. doi: 10.1016/j.jbc.2023.105431 (PMC10704377; doi:10.1016/j.jbc.2023.105431)
Supplement: Supporting Figure legends S1–S7 and Table S1 legend [file mmc1.doc]

***Supplementary MATERIALS***

**Supplementary Figure legends**

**Table S1. Table showing a list of oligomers used in the study.**

**Figure S1. *In silico* analysis of *c-MYC* exon 1/intron 1 breakpoint clusters (Cluster II). A.** Mapping of reported *c-MYC* breakpoints Cluster I and II. P1 and P2 are promoters. 143 patient breakpoints of t(8;14) present in Burkitt’s lymphoma were mapped onto human chromosome 8 genomic contig, GRCh37.p10 Primary Assembly (NCBI Reference Sequence: NT_008046.16). Yellow color indicates a single breakpoint between two nucleotides, red indicates 2 breakpoints at the same site, blue indicates 2 breakpoints in the span of three nucleotides, whereas yellow and cyan represent 2 breakpoints in the span of four nucleotides. **B.** Schematic representation of the breakpoint region (934 bp) in cluster II covering portions of exon 1 and intron 1 used for this study is labelled as Region 4 (indicated in red box). Region 4 is subdivided into three overlapping regions, namely Region 1 (350 bp), Region 2 (331 bp), and Region 3 (293 bp). P0, P1, P2 and P3 are promoters. Region 4 contained 66 patient breakpoints and was used for further studies. **C.** *In silico* analysis of breakpoint region (Region 4) containing Region 1, 2, and 3 for % breakpoints, GC content and non-B DNA structure motifs, AID binding motifs WRC motifs (where W = A/T, R = purine, and C = cytosine), etc. Non-B DB v2 was used for the analysis of potential non-B DNA motifs.

**Figure S2. Bisulphite modification assay to evaluate single-strandedness at *c-MYC* exon 1/intron 1 breakpoint clusters (Cluster II). A.** Schematic representation of the 934 bp breakpoint region covering portions of exon 1 and intron 1 used for the study (Region 4), which was subdivided into Region 1, Region 2 and Region 3. **B.** Schematic representation of the primer binding site of RST1 and SD28, which amplifies extended Region 1 and overlapped sequences from Region 2 (amplicon size of 478 bp). **C.** Representation showing the distribution of bisulphite converted cytosines for top strand of extended Region 1 of 934 bp breakpoint region. The filled black circle indicates the conversion of cytosine to uracil, while the open circle indicates no conversion. Out of 48 clones sequenced, 26 were from the top strand and represented in the figure. **D.** Schematic representation of three different possible ways for the formation of R-loop structure in the anti-physiological orientation of extended Region 1. Nucleotide positions are marked. RNAP is RNA polymerase. Progression of RNAP on the top strand depicts transcription of c-MYC in anti-physiological orientation resulting in the formation of the displaced bottom strand (template) and, thus, the formation of the R-loop. R-loop and G-quadruplex can be formed simultaneously, resulting in shorter R-loops. The top strand (non-template) strand is represented in orange color, the bottom strand (template strand) is in cyan color, and the nascent RNA is in purple colour. **E.** Schematic representation for the primer binding site of RST3 and RST4 (amplicon size of 321 bp) in *c-MYC* Region 2. **F.** Bisulphite sensitivity for the top strand of 331 bp present from Region 2. The filled circle indicates the conversion of cytosine to uracil, while the open circle indicates no conversion. CpG sites are marked with an open red circle. A total of 110 clones were sequenced for the region, of which 40 were from the top strand. 30 randomly selected clones are represented in the figure. **G.** Representative bisulphite sensitivity for the bottom strand of 331 bp region spanning Region 2. The filled circle indicates the conversion of cytosine to uracil, while the open circle indicates no conversion. Such cytosine conversion was not seen in the top strand of the 331 bp fragment of Region 2 in genomic DNA. 110 clones were sequenced using primer sets RST3 and RST4, of which 70 were from the bottom strand, and 10 selected clones with maximum conversions are shown. **H.** Schematic representation for primer binding sites in *c-MYC* Region 4. In this case, region 2 is amplified using primer sets of SD26 and SD27 (amplicon size of 414 bp).  **I.** Representative bisulphite sensitivity for the top strand of 414 bp region spanning Region 2. The filled circle indicates the conversion of cytosine to uracil, while the open circle indicates no conversion. Each row of circles represents a single DNA molecule (a clone). A total of 56 clones were sequenced for Region 2 using primer sets SD26 and SD27, of which 31 were from the top strand, and 10 representative clones with maximum conversions are shown in the image. **J.** Schematic representation of bisulfite converted clones, wherein each horizontal line represents stretch conversion regions in a molecule with vertical bars representing converted cytosine when analysed from plasmid DNA. CpG sites are marked with a red circle, CpG sites present after four continuous conversions are considered as converted cytosines and marked with a filled red circle, while an open red circle indicates a CpG site without conversion in panels C, F, G, and I. A single DNA molecule (a clone) is represented in each row in panels C, F, G, and I.

**Figure S3. Analysis of single-stranded regions in c-*MYC* gene of Raji cells following treatment with actinomycin D. A.** Schematic representation showing the primer binding site of NK39 and NK38, which amplifies extended Region 1 covering the upstream sequence of Region 1 (light green) and overlapped sequences from Region 2 (688 bp). **B.** Representation showing converted cytosines following treatment with bisulphite from a bottom strand of extended region 1 (688 bp). Raji cells were incubated with actinomycin D (0.5 μM) for 12 h, and genomic DNA was isolated and subjected to bisulphite modification assay. Extended Region 1 was PCR amplified, TA cloned, and sequenced. Out of 60 clones analysed, 25 clones were from the top strand. The filled black circle indicates the conversion of cytosine to uracil, while the open circle indicates no conversion. **C.** Representation of bisulphite converted cytosine for the top strand of 688 bp region extended Region 1 (35 clones). **D.** Bar graph showing the number of clones containing continuous stretches of cytosine conversion (<10) in the bottom strand when analyzed from Raji cells. No clones had continuous cytosine conversion in actinomycin D-treated clones, and hence 0.1 was taken as an arbitrary number to plot the graph. **E.** Bar graph representing the number of clones containing 10 continuous cytosine conversions in the top strand. For both graphs, zero clones had more than 10 continuous cytosine conversions, and hence 0.1 was taken as an arbitrary number to plot the graph. CpG sites are marked with a red circle, CpG sites present after four continuous conversions are considered converted cytosines and marked with a filled red circle, while an open red circle indicates a CpG site without conversion in panels B and C. A single DNA molecule (a clone) is represented in each row in panels B and C.

**Figure S4**. **Bisulphite conversions on template and nontemplate strand of pKD2.** **A.** Cumulative cytosine conversions of the nontemplate strand (top strand) from all the bisulfite clones sequenced. The pink box represents the area of stretch conversion. **B.** Cytosine conversions of template strand (bottom strand) for single molecules are shown. Following the transcription of pKD2, products were bisulphite treated, desulphonated and purified. Region 2 was then cloned and sequenced. G1 and G2 refer to the region corresponding to independent G-quadruplex motifs.

**Figure S5. Characterization of G-quadruplexes formed in c-*MYC* breakpoint region. A.** CD spectra for KD16, KD17, KD18 and KD19 in the absence and presence of KCl (100 mM) are presented. KD16 and KD18 are G-rich strands; their complementary C-rich strands are KD17 and KD19, respectively. **B.** CD spectra for KD16 and KD18 at various temperatures 25°C, 55°C, 90°C (denaturation), and after renaturation at 25°C. **C.** KD16, KD27, KD28 and KD29 were incubated in the presence of KCl (100 mM) and resolved in the presence of KCl (100 mM), in the gel and running buffer. KD27, KD28, KD29 are mutants for KD16 oligomer. KD30 and KD31 were incubated in the presence of KCl (100 mM) and resolved in the absence or presence of KCl (100 mM), in the gel and running buffer. KD30 and KD31 are mutants for KD18 oligomer. **D.** The oligomeric sequence of KD16 mutant (KD45) and its complementary KD46 (top panel). KD45 and KD46 were incubated in the presence of KCl (100 mM) and resolved in the absence (left panel) or presence (right panel) of KCl (100 mM), in the gel and running buffer. **E.** CD spectra for KD45 and KD46 in the absence and presence of KCl (100 mM).

**Figure S6. Primer extension on CsCl-EtBr purified pKD2 to determine single-strandedness. 4. A, B.** Primer extension on supercoiled and linearized pKD2 purified by CsCl-EtBr density gradient centrifugation. Primer extension assay was carried out on CsCl-EtBr purified pKD2 (A), and on Nae I linearized pKD2 (B). pDNA (100, 200, 300, 400 ng), incubated in the presence of 50 mM KCl, was used for primer extension studies using radioactively labeled *RST4. The reaction products were resolved on 8% denaturing PAGE, and pause sites are indicated by boxes in the panels.

**Figure S7. Schematic showing the promoters in the antisense strand of *c-MYC* cluster II.** A sequence with the promoters in the antisense strand is shown. E1, E2, and E3 are exons in *MYC*. This sequence includes *MYC* cluster II in the anti-physiological direction with a few bases flanking upstream. Transcription start sites are marked in bold and predicted using the Promoter prediction server. The red arrow represents the promoter predicted.
