# Supplementary material for: Evaluation of potential role of R-loop and G-quadruplex DNA in the fragility of c-MYC during chromosomal translocation associated with Burkitt’s lymphoma
Source: J Biol Chem. 2023 Nov 4;299(12):105431. doi: 10.1016/j.jbc.2023.105431 (PMC10704377; doi:10.1016/j.jbc.2023.105431)
Supplement: Supporting Figures S1–S7 [file mmc2.pdf]

Figure S1A

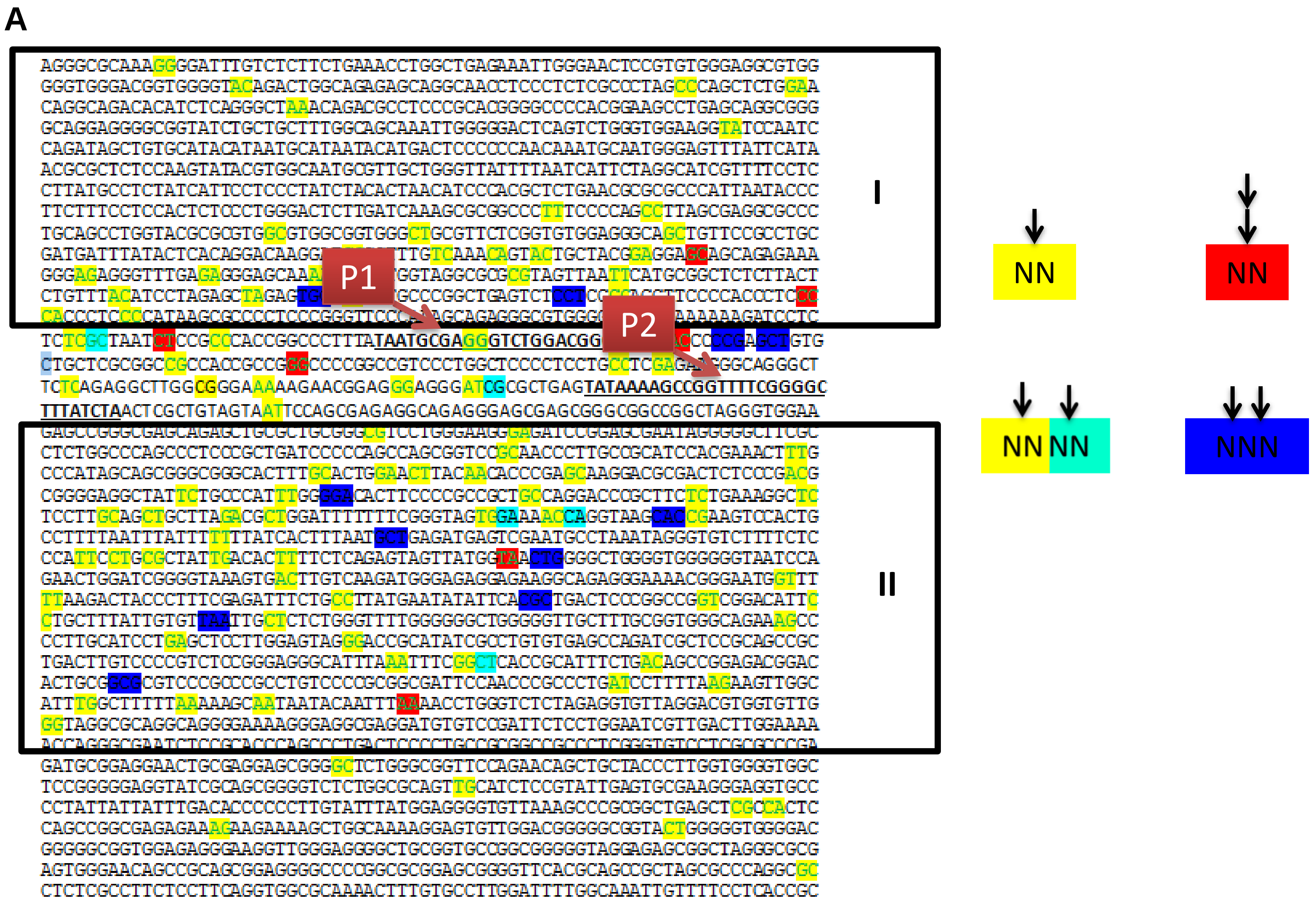

Figure S1B, C

B

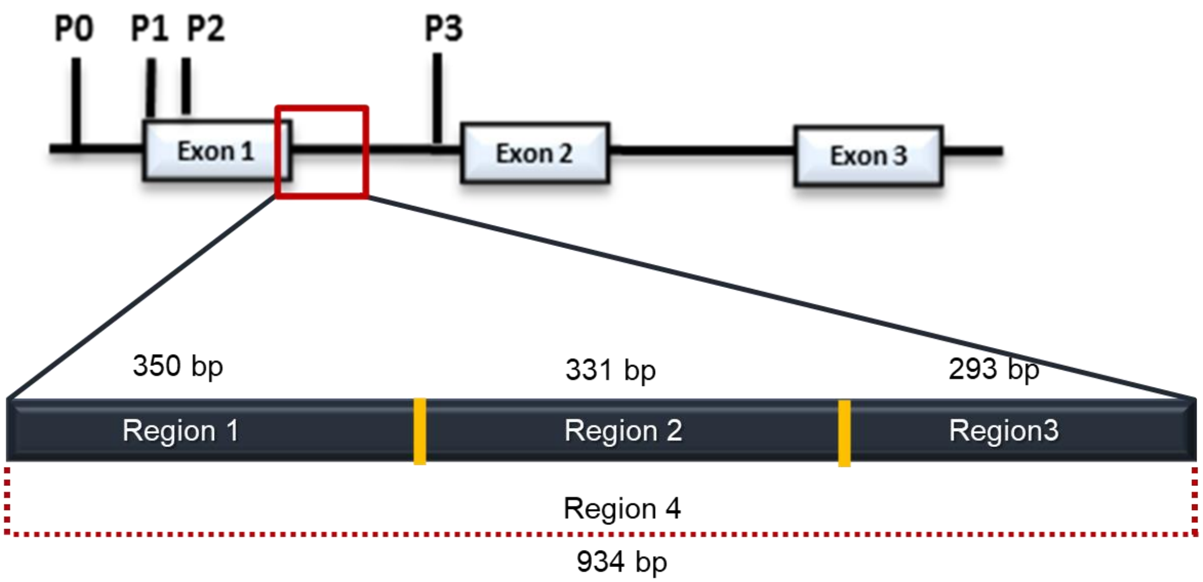

C

Features	Region 1	Region 2	Region 3
% Breakpoints	44	35	26
% GC content	57	50	56
% C (top strand)	32	19	29
% G (top strand)	25	31	27
WRC motif	25	15	11
G-quadruplex forming repeats	Yes	No	No
Z-DNA motifs	No	No	No
Triplex motifs and mirror repeats	No	No	No
Direct repeats and slipped motifs	No	No	No
Inverted repeats and cruciform motifs	No	Yes	Yes

Figure S2

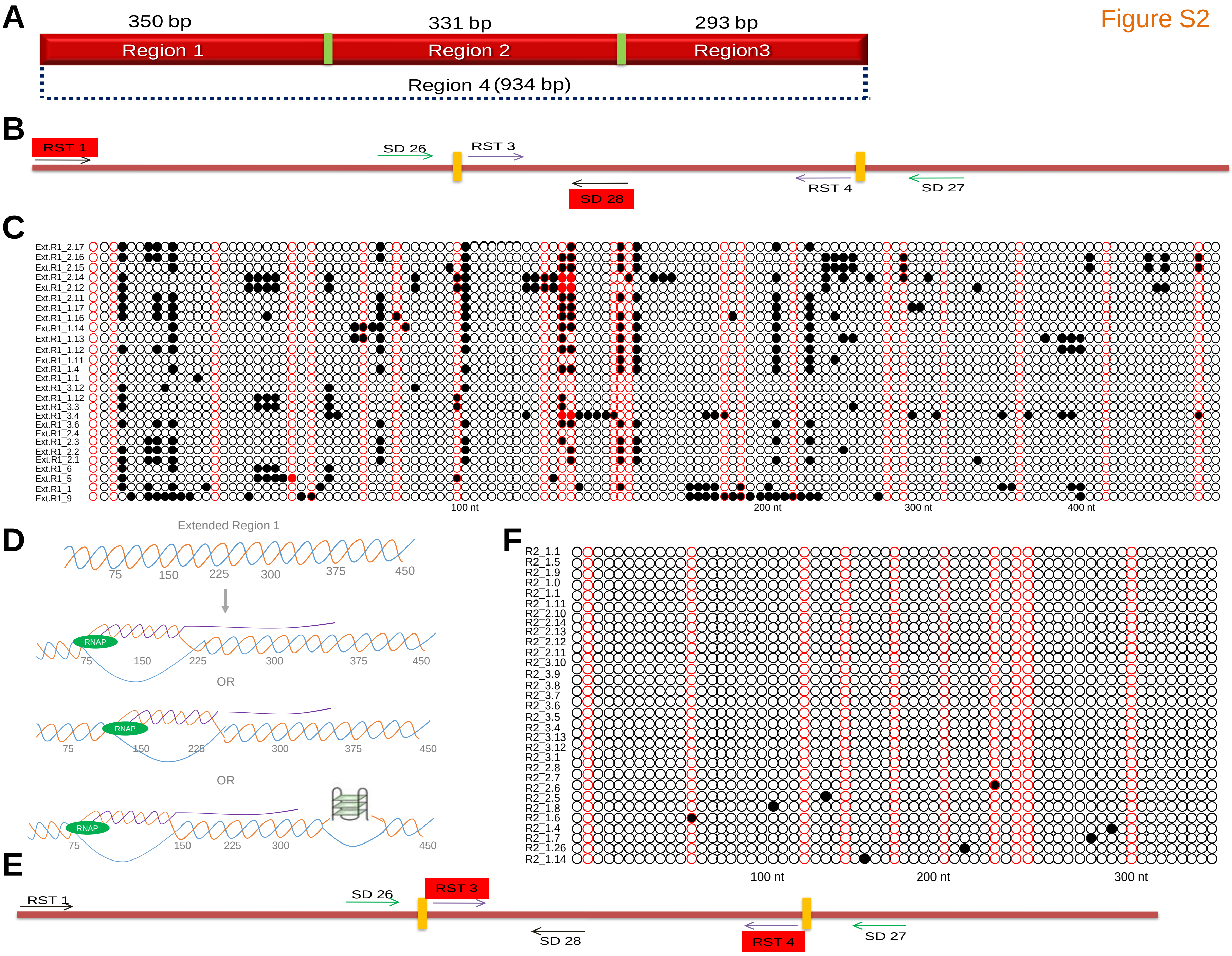
A
350 bp
331 bp
293 bp
Region 1
Region 2
Region3
Region 4 (934 bp)
B
RST 1
SD 26
RST 3
SD 28
RST 4
SD 27
C
Ext.R1_2.17
Ext.R1_2.16
Ext.R1_2.15
Ext.R1_2.14
Ext.R1_2.12
Ext.R1_2.11
Ext.R1_1.17
Ext.R1_1.16
Ext.R1_1.14
Ext.R1_1.13
Ext.R1_1.12
Ext.R1_1.11
Ext.R1_1.4
Ext.R1_1.1
Ext.R1_3.12
Ext.R1_1.12
Ext.R1_3.3
Ext.R1_3.4
Ext.R1_3.6
Ext.R1_2.4
Ext.R1_2.3
Ext.R1_2.2
Ext.R1_2.1
Ext.R1_6
Ext.R1_5
Ext.R1_1
Ext.R1_9
100 nt
200 nt
300 nt
400 nt
D
Extended Region 1
75
150
225
300
375
450
RNAP
OR
E
RST 1
SD 26
RST 3
SD 28
RST 4
SD 27
F
R2_1.1
R2_1.5
R2_1.9
R2_1.0
R2_1.1
R2_1.11
R2_2.10
R2_2.14
R2_2.13
R2_2.12
R2_2.11
R2_3.10
R2_3.9
R2_3.8
R2_3.7
R2_3.6
R2_3.5
R2_3.4
R2_3.13
R2_3.12
R2_3.1
R2_2.8
R2_2.7
R2_2.6
R2_2.5
R2_1.8
R2_1.6
R2_1.4
R2_1.7
R2_1.26
R2_1.14
100 nt
200 nt
300 nt

G

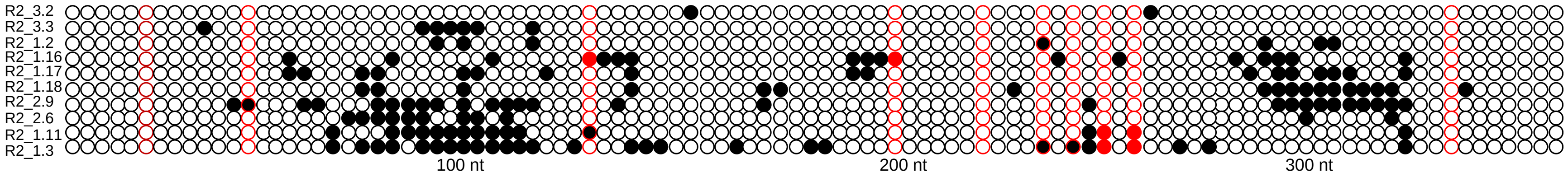
R2_3.2
R2_3.3
R2_1.2
R2_1.16
R2_1.17
R2_1.18
R2_2.9
R2_2.6
R2_1.11
R2_1.3
100 nt
200 nt
300 nt

H

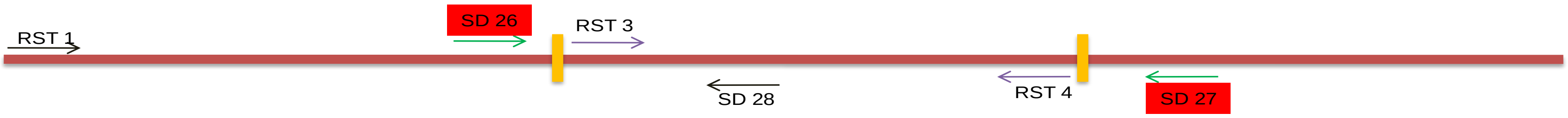
SD 26
RST 1
RST 3
SD 28
RST 4
SD 27

I

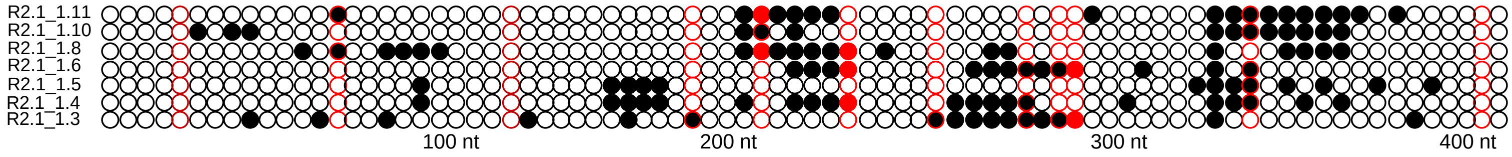
R2.1_1.11
R2.1_1.10
R2.1_1.8
R2.1_1.6
R2.1_1.5
R2.1_1.4
R2.1_1.3
100 nt
200 nt
300 nt
400 nt

J

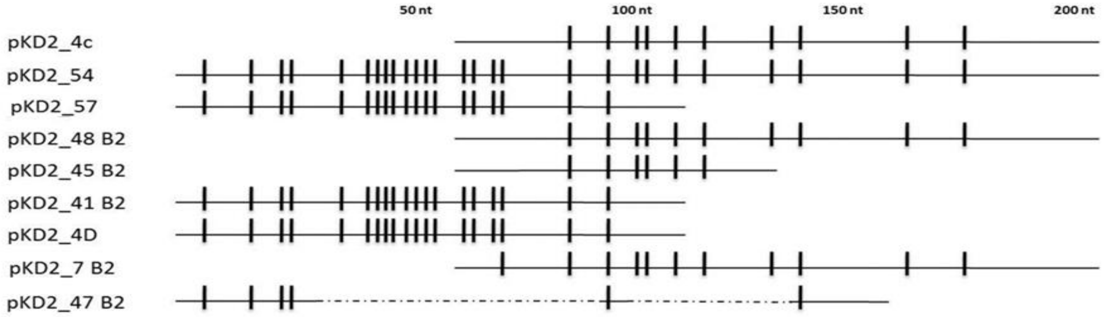
50 nt
100 nt
150 nt
200 nt
pKD2_4c
pKD2_54
pKD2_57
pKD2_48 B2
pKD2_45 B2
pKD2_41 B2
pKD2_4D
pKD2_7 B2
pKD2_47 B2

Figure S3

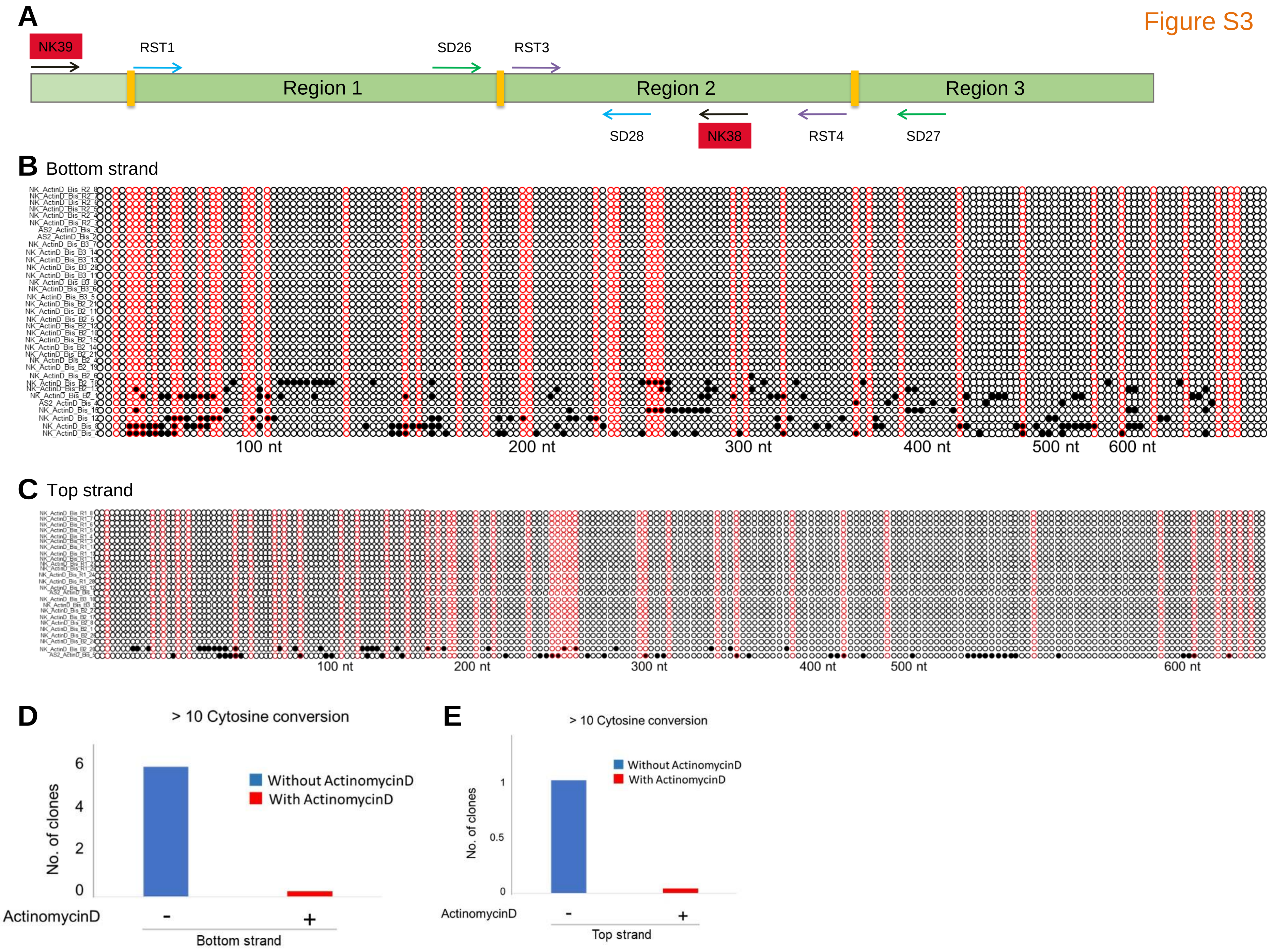
A
NK39
RST1
SD26
RST3
Region 1
Region 2
Region 3
SD28
NK38
RST4
SD27
B Bottom strand
100 nt
200 nt
300 nt
400 nt
500 nt
600 nt
C Top strand
100 nt
200 nt
300 nt
400 nt
500 nt
600 nt
D
> 10 Cytosine conversion
No. of clones
Without ActinomycinD
With ActinomycinD
ActinomycinD
-
+
Bottom strand
E
> 10 Cytosine conversion
No. of clones
Without ActinomycinD
With ActinomycinD
ActinomycinD
-
+
Top strand

Figure S4

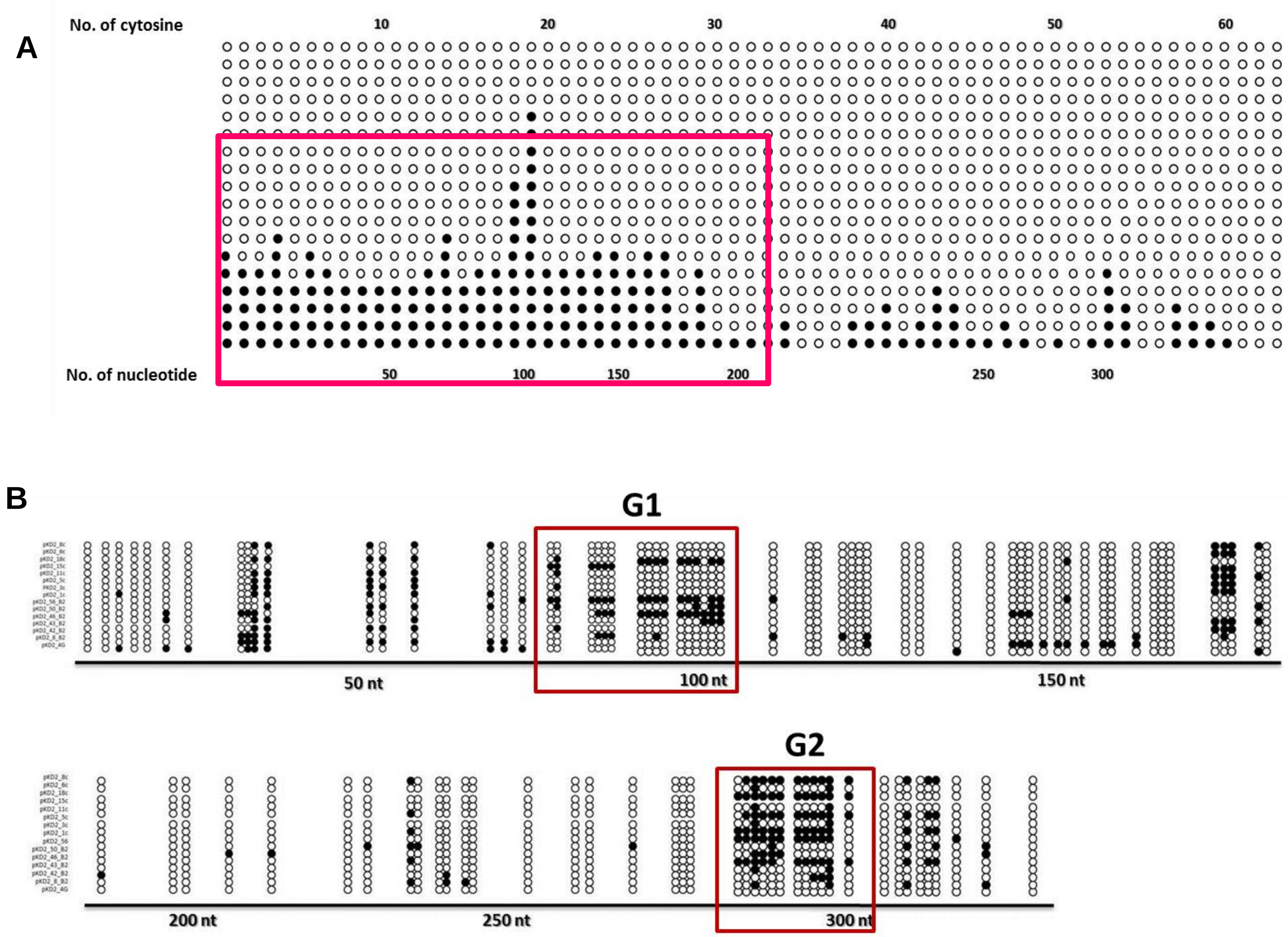
A
No. of cytosine
10
20
30
40
50
60
No. of nucleotide
50
100
150
200
250
300
B
G1
50 nt
100 nt
150 nt
G2
200 nt
250 nt
300 nt

Figure S5

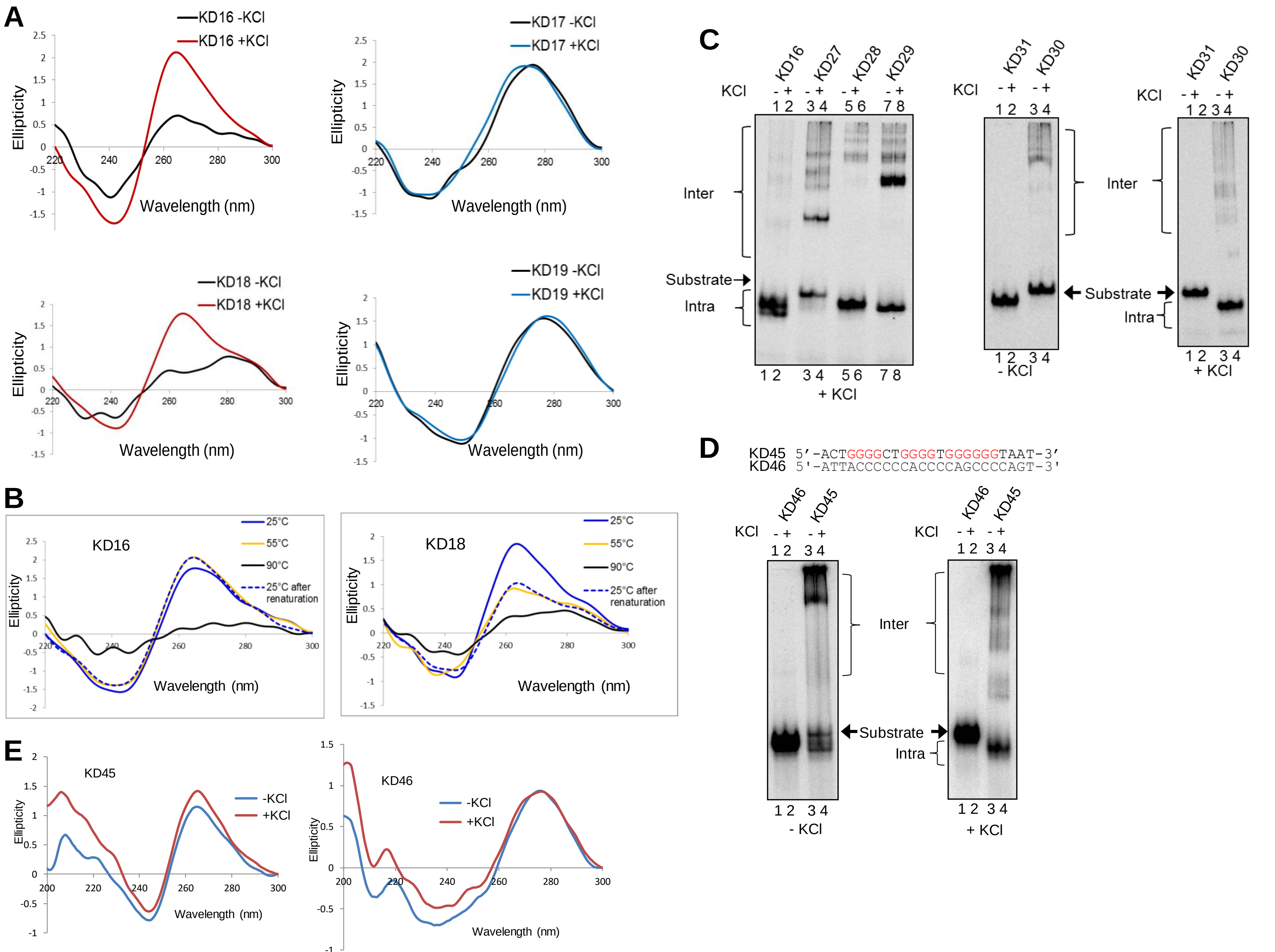
A
KD16 -KCl
KD16 +KCl
KD17 -KCl
KD17 +KCl
KD18 -KCl
KD18 +KCl
KD19 -KCl
KD19 +KCl
Ellipticity
Wavelength (nm)
B
KD16
KD18
25°C
55°C
90°C
25°C after renaturation
C
KCl
KD16 KD27 KD28 KD29
KD31 KD30
- + - + - + - +
1 2 3 4 5 6 7 8
Inter
Substrate
Intra
+ KCl
- KCl
D
KD45 5'-ACTGGGGCTGGGGTGGGGGGTAAT-3'
KD46 5'-ATTACCCCCCACCCCAGCCCCAGT-3'
KD46 KD45
E
KD45
KD46
-KCl
+KCl

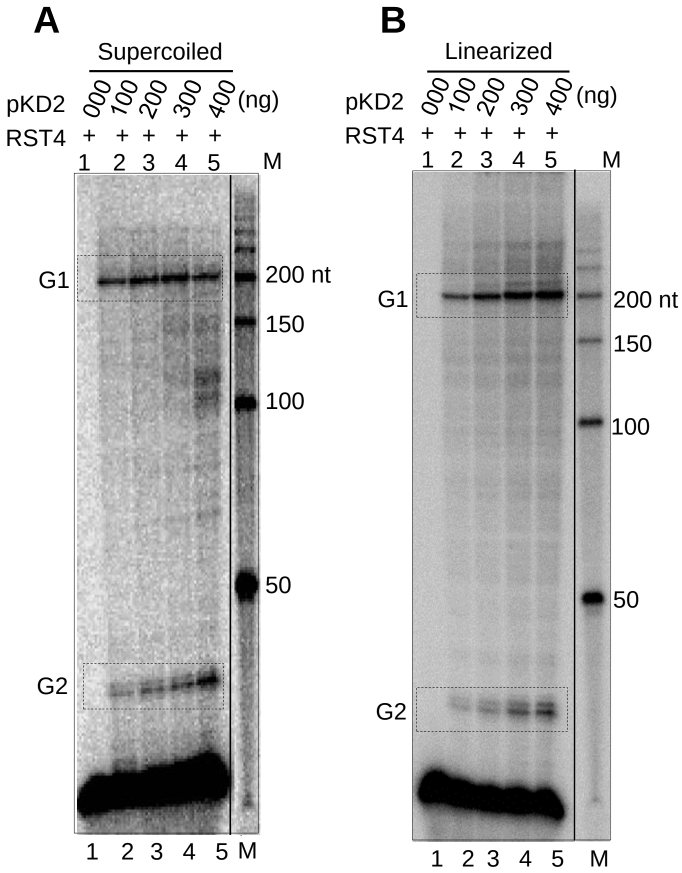
A
Supercoiled
pKD2 000 100 200 300 400 (ng)
RST4 + + + + +
1 2 3 4 5 M
G1
G2
200 nt
150
100
50
1 2 3 4 5 M
B
Linearized
pKD2 000 100 200 300 400 (ng)
RST4 + + + + +
1 2 3 4 5 M
G1
G2
200 nt
150
100
50
1 2 3 4 5 M

Figure S7

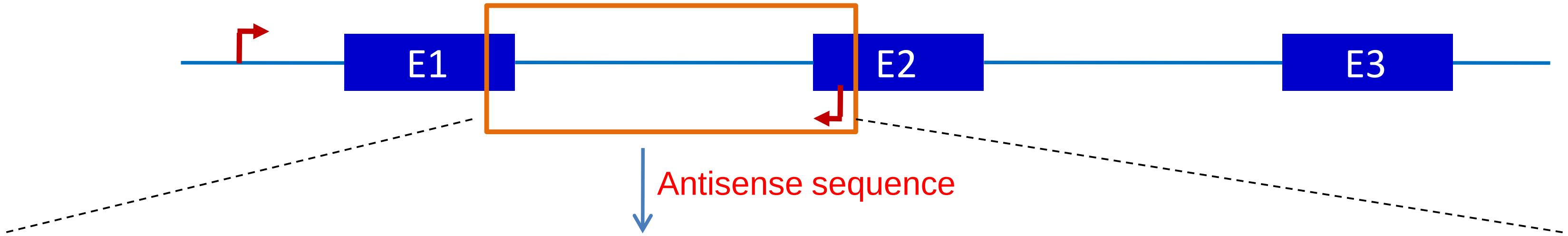

GCAGTCTTGAGTTAAAGGGGTCTTGGTGCGGAAACCTGGCGCAGCGCGCAGTGCGCGCCACAGTCCCGAACCTCTCCCCTTGCAGAGCTATCCCCTAAAGCGGCTG

GGTGGTCTTGGTGGGGGAATAAAGGGAGCACCCTTTCACCCCCTTTG**G**ACAGTCCCCTGCTATCTCGGAGACGCACTTAGTGAACCAGCGGCTTGGTGCCCGCCGA

GCCCCCGCTCCCCCGGGAGCCCGGAGCGCAAAGCCCGGGAGTCGGCCCCGCAGCGGCAGAGGAATCGAAATCGGCCCTGGCGCCCTTAAGAAGCCGCGGGAGGTGG

CGGTGAGGAAAACAATTTGCCAAAATCCAAGGCACAAAGTTTTGCGCCACCTGAAGGAGAAGGCGAGAGGCGCCTGGGCGCTAGCGGCTGCGTGAACCCCGCTCCG

CGCCGGGGCCCCTCCGCTGCGGCTGTTCCCACTCGCGCCCTAGCCGCTCTCCTACCCCCGCCGGCACCGCAGCCCCTCCCAACCTTCCCTCTCCACCGCCCCCGTC

CCCACCCCCAGTACCGCCCCCGTCCAACACTCCTTTTGCCAGCTTTTCTTCTTTCTCTCGCCGGCTGGAGTGGCGAGCTCAGCCGCGGGCTTTAACACCCCTCCAT

AAATACAAGGGGGGTGTCAAATAATAATA**G**GGGCACCTCCCTTCGCACTC**A**ATACGGAGATGCAACTGCGCCAGAGACCCCGCTGCGATACCTCCCCCGGAGCCA

CCCCACCAAGGGTAGCAGCTGTTCTGGAACCGCCCAGAGCCCCGCTCCTCGCAGTTCCTCCGCATCTCGGGCGCGAGGACACCCGAGGGCGGCCGCGGCAGGGGAG

TCAGGGCTGGGTGCGGAGATTCGCCCTGGTTTTTCCAAGTCAACGATTCCAGGAGAATCGGACACATCCTCGCCTCCCTTTTCCCCTGCCTGCGCCTACCCAACAC

CACGTCCTAACACCTCTAGAGACCCAGGTTTTAAATTGTATTATTGCTTTTTAAAAAGCCAAATGCCAACTTCTTAAAA**G**GATCAGGGCGGGTTGGAATCGC**C**GCG

GGGACAGGCGGGCGGGACGCGCCGCAGTGTCCGTCTCCGGCTGTCAGAAATGCGGTGAGCCGAAATTTAAATGCCCTCCCGGAGACGGGGACAAGTC**A**GCGGCTGC

GGAGCGATCTGGCTCACACAGGCGATATGCGGTCCCTACTCCAAGGAGCTCAGGATGCAAGGGGCTTTCTGCCCACCGCAAAGCAACCCCCAGCCCCCCAAAACCC

AGAGAGCAATTAACACAATAAAGCAGGAATGTCCGACCGGCCGGGAGTCAGCGTGAATATATTCATAAGGCAGAAATCTCGAAAGGGTAGTCTTAAAAACCATTCC

CGTTTTCCCTCTGCCTTCTCCTCTCCCATCTTGACAAGTCACTTTACCCCGATCCAGTTCTGGATTACCCCCCACCCCAGCCCCAGTTACCATAACTACTCTGAGA

AAAGTGTCAATAGCGCAGGAATGGGAGAAAAGACACCCTATTTAGGCATTCGACTCATCTCAGCATTAAAGTGATAAAAAAATAAATTAAAAGGCAAGTGGACTTC

GGTGCTTACCTGGTTTTCCACTACCCGAAAAAAATCCAGCGTCTAAGCAGCTGCAAGG**A**GAGCCTTTCAGAGAAGCGGGTCCTGGCAGCGGCGGGGAAGTGTCCCC

AAATGGGCAGAATAGCCTCCCCGCGTCGGGAGAGTCGCGTCCTTGCTCGGGTGTTGTAAGTTCCAGTGCAAAGTGCCCGCCCGCTGCTATGGGCAAAGTTTCGTGG

ATGCGGCAAGGGTTGCGGACCGCTGGCTGGGGGATCAGCGGGAGGGCTGGGCCAGAGGCGAAGCCCCCTATTCGCTCCGGATCTCCCTTCCCAGGACGCCCGCAGC

GCAGCTCTGCTCGCCCGGCTCTTCCACCCTAGCCGGCCGCCCGCTCGCTCCCTCTGCCTCTCGCTGGAATTACTACAGCGAGTTAGATAAAGCCCCGAAAACCG
