# Supplementary material for: Evaluation of potential role of R-loop and G-quadruplex DNA in the fragility of c-MYC during chromosomal translocation associated with Burkitt’s lymphoma
Source: J Biol Chem. 2023 Nov 4;299(12):105431. doi: 10.1016/j.jbc.2023.105431 (PMC10704377; doi:10.1016/j.jbc.2023.105431)
Supplement: Supporting Table S1 [file mmc3.doc]

| S.N. | Oligomer | Sequence |
| --- | --- | --- |
| 1. | RST1 | 5’-GAGCGAATAGGGGGCTTC-3’ |
| 2. | RST2 | 5’-GCATTCGACTCATCTCAGCA -3’ |
| 3. | RST3 | 5’-TGCTGAGATGAGTCGA-ATGC-3’ |
| 4. | RST4 | 5’-GGATGCAAGG-GGCTTTCTGC-3’ |
| 5. | KD16 | 5’-ACTGGGGCTGGGGTGGGGGGTAATCCAGAACTGGATCGGGGT-3’ |
| 6. | KD17 | 5’-ACCCCGATCCAGTTCTGGATTACCCCCCACCCCAGCCCCAGT-3’ |
| 7. | KD18 | 5’-TCTGGGTTTTGGGGGGCTGGGGGTTGCTTTGCGGTGGGCA-3’ |
| 8. | KD19 | 5’-TGCCCACCGCAAAGCAACCCCCAGCCCCCCAAAACCCAGA-3’ |
| 9. | KD27 | 5’-ACTGCATCTGGGGTGGGGGGTAATCCAGAACTGGATCGGGGT-3’ |
| 10. | KD28 | 5’-ACTGGGGCTGGGGTGCATGGTAATCCAGAACTGGATCGGGGT-3 |
| 11. | KD29 | 5’-ACTGGGGCTGGGGTGGGGGGTAATCCAGAACTGGATCCATGT-3’ |
| 12. | KD30 | 5’-TTGGGGGGCTGGGGGTTGCTTTGCGGTGGGCA-3’ |
| 13. | KD31 | 5'-TGCCCACCGCA-AAGCAACCCCCAGCCCCCCAA-3’ |
| 14. | SD26 | 5'- ACCAGGTAAGCACCGAAGT-3' |
| 15. | SD27 | 5'- ATATGCGGTCCCTACTCCAA-3' |
| 16. | SD28 | 5'- TCCCATCTTGACAAGTCACT-3' |
| 17. | ET50 | 5'- GGTCTTCAATGTGATGTTCAATGTATA-3' |
| 18. | RBK17 | 5’-GCATTGTTCGTTACATGGTCC-3’ |
| 19. | RBK18 | 5’-GTGGAAGCGGATGAGTAAGAAG-3’ |
| 20. | RBK21 | 5’-CTAACCATCTTCTCCTTACACCTAG-3’ |
| 21. | RBK22 | 5’-GTTTGCTAATACAATGCCAGTCAG G-3’ |
| 22. | NK 33 | 5’-AGTGACTTGTCAAGATGGGA-3’ |
| 23. | NK 34 | 5’-AAAGCAGGAATGTCCGACCG-3’ |
| 24. | NK 35 | 5’-CAGAAAGCCCCTTGCATCC-3’ |
| 25. | NK 36 | 5’ -GGAGACGGGGACAAGTCAG-3’ |
| 26. | NK 38 | 5’-AGCAGGAATGTCCGACCG 3’ |
| 27. | NK 39 | 5’-GTAATTCCAGCGAGAGGC-3’ |
| 28. | SV 54 | 5’-ACACAGTCCAGACACTCTGC-3’ |
| 29. | SV 55 | 5’-ACGTGCAGAAACTCCTTGTTC-3’ |
| 30. | SV 62 | 5’-GGCTCCTGACAGACGGG-3’ |
| 31. | SV 63 | 5’-GCATGGGCTCCGTCC-3’ |
| 32. | SV 64 | 5’-GGACGGAGCCCATGC-3’ |
| 33. | SV 65 | 5’-CGTATTGCTGCCGCCT-3’ |
